# MDE and LLM Synergy for Network Experimentation: Case Analysis of Wireless System Performance in Beaulieu-Xie Fading and κ-µ Co-Channel Interference Environment with Diversity Combining †

**DOI:** 10.3390/s24103037

**Published:** 2024-05-10

**Authors:** Dragana Krstic, Suad Suljovic, Goran Djordjevic, Nenad Petrovic, Dejan Milic

**Affiliations:** 1 Faculty of Electronic Engineering, University of Nis, 18000 Nis, Serbia; nenad.petrovic@elfak.ni.ac.rs (N.P.); dejan.milic@elfak.ni.ac.rs (D.M.); 2 Academy of Technical Professional Studies, 11120 Belgrade, Serbia; ssuljovic@atssb.edu.rs (S.S.); gdjordjevic@atssb.edu.rs (G.D.)

**Keywords:** Beaulieu-Xie fading, κ-µ co-channel interference, first-order performance, second-order performance, selection combining, large language model, model-driven engineering

## Abstract

Channel modeling is a first step towards the successful projecting of any wireless communication system. Hence, in this paper, we analyze the performance at the output of a multi-branch selection combining (SC) diversity receiver in a wireless environment that has been distracted by fading and co-channel interference (CCI), whereby the fading is modelled by newer Beaulieu-Xie (BX) distribution, and the CCI is modelled by the κ-µ distribution. The BX distribution provides the ability to include in consideration any number of line-of-sight (LOS) useful signal components and non-LOS (NLOS) useful signal components. This distribution contains characteristics of some other fading models thanks to its flexible fading parameters, which also applies to the κ-µ distribution. We derived here the expressions for the probability density function (PDF) and cumulative distribution function (CDF) for the output signal-to-co-channel interference ratio (SIR). After that, other performances are obtained, namely: outage probability (Pout), channel capacity (CC), moment-generating function (MGF), average bit error probability (ABEP), level crossing rate (LCR), and average fade duration (AFD). Numerical results are presented in several graphs versus the SIR for different values of fading and CCI parameters, as well as the number of input branches in the SC receiver. Then, the impact of parameters on all performance is checked. From our numerical results, it is possible to directly obtain the performance for all derived and displayed quantities for cases of previously known distributions of fading and CCI by inserting the appropriate parameter values. In the second part of the paper, a workflow for automated network experimentation relying on the synergy of Large Language Models (LLMs) and model-driven engineering (MDE) is presented, while the previously derived expressions are used for evaluation. Due to the aforementioned, the biggest value of the obtained results is the applicability to the cases of a large number of other distributions for fading and CCI by replacing the corresponding parameters in the formulas for the respective performances.

## 1. Introduction

Channel modeling is a first step towards the successful projecting of any wireless communication system. The wireless environment can be distracted by fading and co-channel interference (CCI) [1]. To describe the wireless channels as better as possible, many measurements should be carried out in the environment. After these measurements, it is essential to create as adequate models as possible.

For that purpose, Beaulieu and Xie Jiandong defined a new distribution model, called the Beaulieu-Xie (BX), which is suitable to successfully describe fading that contains an arbitrary number of both, the line-of-sight (LOS) and the non-LOS (NLOS) components of the useful signal [2]. This mathematical model contains the features of other fading distributions due to three parameters that characterize BX distribution (*m*, λ, and Ω). 

Because of the above, the BX distribution is used for modeling fading in densely packed small cells called femtocells, for millimeter (mmWave) and terahertz (THz) communication systems, as well as for short-range 6G random-access channels, when there are multiple reflected signals. In any case, for now, BX distribution has its greatest application in signal propagation in small buildings and fast-moving trains.

The BX fading model is a general model. Other models of known fading distributions are included in the BX model: κ-µ, non-central chi, generalized Rician, and others which can be obtained from it: Rician distribution, one-sided Gaussian distribution, Rayleigh and Nakagami-*m* distribution. By approximation, a log-normal distribution can also be derived from the κ-µ distribution. It was shown that κ-µ distribution is in quite good agreement with the experimentally obtained data when it was revealed [3]. It was established by measurements at that time that the Nakagami-*m* fading model is almost ideally suited only to model wireless channels with scattered fading components, but it became clear that the Nakagami-*m* model cannot describe wireless channels with LOS components. The Ricean fading model is a usual single model for a presentation signal composed of several scattered components and only one LOS component. It was obvious that none of the abovementioned distributions are suitable for modeling the fading channel where multiple dominant specular components are present in addition to the diffuse scatter power. Therefore, it was necessary to construct a new model that would overcome this deficiency by including several direct components [2]. Thus, a BX model is defined to include not only one LOS component but more LOS and NLOS components.

Recently, some groups of authors have paid attention to the performance over the BX fading channel and analyzed it under different transmission conditions. Kansal and Singh analyzed effective capacity of the BX fading channel for SISO systems in [4]; the capacity of this channel with a maximal ratio combining (MRC) receiver in [5]; the average bit error rate (ABER) of binary phase-shift keying (BPSK) and differential phase-shift keying (DPSK) in [6]; the ABER and the outage probability (Pout) of a selection combining (SC) receiver in [7]; the average symbol error probability (ASEP) of M-ary differential phase-shift keying (MDPSK), non-coherent M-ary frequency shift keying (MFSK), and coherent M-ary phase-shift keying (MPSK) in [8]; and the ASEP of generalized M-ary quadrature amplitude modulation (M-QAM) in [9].

Performance analysis of a dual-branch switch-and-stay combining (SSC) diversity receiver for a BX fading model is presented in [10]. The expressions of probability density function (PDF), cumulative distribution function (CDF), and moment-generating function (MGF) are derived. Based on them, the expressions for moments, average output signal-to-noise ratio (SNR), channel capacity (CC), Pout, and ABER for a binary modulation scenario are derived. In [11], the performance of femtocells with an MRC diversity receiver is observed. The expressions for Pout, amount of fading (AF), and ASEP for coherent and non-coherent modulations are obtained in the closed form for the BX channel. Also, CC is evaluated. 

For the time being, the team consisting of the members Olutayo, Cheng, and Holzman has intensively analyzed the BX channel [12,13,14,15]. For an *L*-branch SC receiver over BX fading channels with arbitrary correlation, the Pout and error rate performance are derived in closed-form expression in [12], while in [13], the same performances are derived for the case with an equal-gain combining (EGC) receiver. For a BX fading channel using different diversity combining schemes with different diversity orders, SC, EGC, and MRC, asymptotically tight upper and lower bounds for the Pout and error rate performance for high signal-to-noise ratios are performed in [14]. This group of authors analyzed the level crossing rate (LCR) and average fade duration (AFD) for the BX fading model and a diversity scheme using MRC in [15]. The derived measures of the BX fading model showed improvements tied to the performance of the Ricean and Nakagami-*m* fading models. Finally, Olutayo united the previously published results of research into the performance of wireless systems with BX fading in her doctoral dissertation [16].

In most of the works, diversity combining techniques were used to reduce the effect of fading. Diversity mitigates the effects of fading by combining multiple independent fading paths, since the chance that all the branches are in deep fade at the same time is very low [1]. 

The diversity combining techniques have different levels of complexity and performance. A few of them are used in wireless systems the most often. Among them are the ones mentioned above: MRC, EGC, and SC combiners. The MRC provides the best diversity performance because it combines fading paths optimally. In this scheme, all paths are co-phased and summed with optimal weighting to maximize output SNR or SIR. Here, the standard ABER is not easy to obtain in closed form since the integral in the ABER formula often diverges. So, analysis of the MRC is simplified using the MGF approach. The EGC is simpler than MRC. In this method, paths are co-phased and summed but with equal gain. This type of combining is easier to implement compared to MRC. 

With selection diversity, the receiver selects the antenna with the highest received signal power, or output signal-to-co-channel interference ratio (SIR), and ignores data from the other antennas. The chosen receiver antenna is one which gives maximal SIR. Since at each moment only one antenna is observed, no co-phasing is required.

The CCI also exists in wireless systems and has to be accounted for in calculating their performance. The CCI represents crosstalk from two different radio transmitters using the same channel. This is a phenomenon where the signal transmitted in one channel of a wireless communication system produces an undesired impact on another channel. Thus, the CCI is narrowly tied up with frequency reuse when the same frequency band is used by two or more base stations that are located in the vicinity of each other. If the distance between the cells that use the same frequency band increases, the possibilities for crosstalk in wireless communication due to frequency reuse decreases. The cellular structure is designed to provide maximal protection against CCI, but it cannot be totally eliminated. That is why CCI can be the dominant factor in determining the system performance, and understanding the influence of CCI on the system performance is of great importance in wireless system design. The CCI can be caused by many other factors, like: poor radiation from antenna side lobs, faulty filtering, bad weather conditions, insufficient cross-polarization isolation, nonlinearity of power amplifier, and so on, but it may be mitigated, like fading, by using diversity combining schemes. 

As far as we know, the CCI, which also deadens wireless systems beside the BX fading, was not taken into account in the available literature until our group of authors. We introduced here the influence of CCI with κ−µ distribution. 

In the twentieth century, Nakagami-*m* was a very popular distribution because of its ease of manipulation and wide range of applicability [3]. However, it was found that in some environments, Rician and even Weibull distributions give better results. By measurements, it was discovered that the tails of the Nakagami-*m* distribution do not fit well to experimental data but only around the mean or median. At the beginning of this millennium, a new fading distribution was proposed—the κ-µ distribution, more flexible than distributions known till that time. Additionally, the κ-µ distribution is a general fading distribution that includes almost all previously known distributions as special cases.

According to that, we included the CCI with a κ-µ distribution that runs in addition to BX fading and derived different performances for these disturbances when attenuated by a multi-branch SC receiver. The SC combiner was chosen for its simplicity, satisfactory performance, and affordability, although EGC and MRC combiners give slightly better performance.

For such a scenario, we derived here the expressions for the PDF and CDF for the output SIR. After, the other performance elements are obtained, as follows: outage probability, level crossing rate, average fade duration, channel capacity, moment-generating function, and ABEP. Numerical results are presented in more graphs versus the SIR for different values of fading and CCI parameters. Then, the impact of parameters on all performance is checked. Finally, the expression is derived.

In the second part, we present a workflow whose goal is to make network planning more convenient and faster, making use of model-driven engineering (MDE)—for network model representation and Large Language Models (LLMs)—automated experiment code generation based on textual description. In this context, the expression derived in the first part of the paper is used for evaluation of the presented approach.

The main contributions of this paper are as follows: (1) derivation of the expressions for performance for the *L*-branch SC receiver in the presence of BX fading and κ-µ CCI; (2) graphical presentation of obtained performance in order to examine the impact of fading and CCI parameters to concerned quantities; and (3) presentation of a tool chain for automated network planning experiment generation starting from free-form text, relying on MDE and LLM.

The paper is structured as follows: following the introduction in Section 1 of a description of the papers from the area, in Section 2, the model of an SC receiver is introduced and SIR-based performances of the first order are derived. The graphical presentation and analysis of these performances are also given. In Section 3, the second-order performances are shown. In Section 4, the proposed network experimentation workflow leveraging MDE and LLMs is described, focusing on experiment generation and verification based on textual descriptions. Finally, Section 5 concludes the paper by giving the main points, highlights advantages and disadvantages, and also mentions future research.

## 2. SIR-Based Performance Analysis 

**Figure 1:**
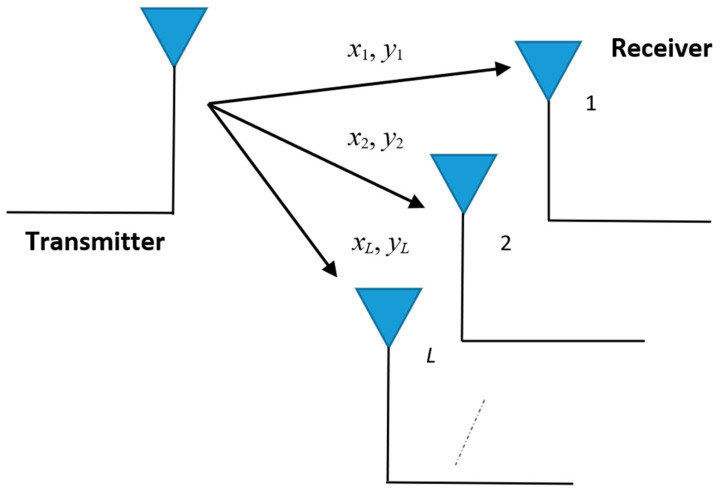
Model of multi-branch SC diversity receiver.

In the next sections, the performance of a wireless system in the presence of BX fading and κ-µ CCI will be determined. In order to mitigate the effects of fading and CCI, a multi-branch SC diversity receiver is used. The model of this receiver is shown in Figure 1. 

The SC receiver operates by feeding the user the signal from the highest value input.

We marked the input signals with: *x_i_*, *i* = 1, 2, …, *L*; *L* ≥ 2, and the output signal with *x*. The input envelopes of CCI are: *y_i_*, *i* = 1, 2, …, *L*, with output value *y*. Given the presence of CCI, performance will be determined based on the output SIR, denoted by *z* and equal to *max*(*z*_1_, *z*_2_, …, *z_L_*), where *z_i_* are the input SIRs equal to the ratios of the useful signals and the CCIs at the input antennas: *z_i_ = x_i_/y_i_*.

### 2.1. The PDF of the Output SIR

The input signals in the SC diversity receiver have the Beaulieu-Xie PDF ([2], Equation (4)):

(1) $$p_{X_{i}}\left(x_{i}\right)=\frac{{2m_{i}x_{i}}^{m}}{\Omega_{i}\lambda_{i}^{m-1}}e^{-\frac{m_{i}}{\Omega_{i}}\left({x_{i}}^{2}+\lambda_{i}^{2}\right)}I_{m-1}\left(\frac{2m_{i}\lambda_{i}}{\Omega_{i}}x_{i}\right)$$

The parameters *m_i_* and *λ_i_* are the fading severity parameter and non-centrality parameter, in a row, and Ω*_i_* are powers of input signals. For PDF of the BX distribution, *m* controls the shape, Ω defines the spread, and *λ* influences the location and height of the mode [16].

The Rician distribution may be obtained from BX distribution if parameter *m_i_* is equal to one, and Nakagami-*m* distribution will be obtained if non-central parameter *λ_i_* is equal to zero. Further, if non-central parameter *λ_i_* is zero, the Rician distribution becomes a Rayleigh, and also if *m_i_* is equal to one in Nakagami-*m* distribution. This feature of the BX distribution is shown in Figure 1.3 in [16].

For a more practical presentation of the PDF of input signals, we will use a modified Bessel function of the first kind of real order *v*, *I_v_*(*z*), developed in an infinite series ([17], Equation (3)), as it is presented by Formula (2) in our conference paper [18]. Now, the PDF is in the form of a sum:

(2) $$p_{x_{i}}\left(x_{i}\right)=2e^{-\frac{m_{i}}{\Omega_{i}}\left({x_{i}}^{2}+{\lambda_{i}}^{2}\right)}\sum_{j_{1}=0}^{+\infty }\frac{{\lambda_{i}}^{2j_{1}}{x_{i}}^{2j_{1}+2m_{i}-1}}{j_{1}!\Gamma \left(j_{1}+m_{i}\right)}{\left(\frac{m_{i}}{\Omega_{i}}\right)}^{2j_{1}+m_{i}}$$

where the Gamma function is labeled by Γ(*z*) ([19], p. 255).

The parameter *λ* can be expressed using the following formula [2]:

(3) $$\lambda_{i}=\sqrt{\kappa_{x}\Omega_{i}},$$

where κ*_x_* is the *K*-factor of the generalized Rician distribution defined as *s*^2^/*nσ*^2^, and representing the power in the LOS component divided by the power in the scatter components, whereby *x* means that κ refers to the fading. When *K* = *λ*^2^/Ω, *λ*^2^ signs the LOS power, and Ω marks the NLOS power. In that case, the PDF becomes:

(4) $$p_{x_{i}}\left(x_{i}\right)=2e^{-\frac{m_{i}}{\Omega_{i}}\left({x_{i}}^{2}+\kappa_{x_{i}}\Omega_{i}\right)}\sum_{j_{1}=0}^{+\infty }\frac{{\left(\kappa_{x}\Omega_{i}\right)}^{j_{1}}{x_{i}}^{2j_{1}+2m_{i}-1}}{j_{1}!\Gamma \left(j_{1}+m_{i}\right)}{\left(\frac{m_{i}}{\Omega_{i}}\right)}^{2j_{1}+m_{i}}$$

From this formula, it can be seen that the BX fading distribution becomes Rician when the fading parameter *m* is equal to 1 for any value of κ = *K*. Also, it can be further reduced to Rayleigh fading distribution when *m* = 1 and κ= 0.

The CCI appearing here has a κ-µ distribution [20]:

(5) $$p_{y_{i}}\left(y_{i}\right)=\frac{2e^{-\frac{\mu_{i}\left(1+\kappa_{y}\right)}{s_{i}}{y_{i}}^{2}}}{e^{\mu_{i}\kappa_{y}}}\overset{+\infty }{\underset{j_{2}=0}{\sum }}\frac{{\mu_{i}}^{2j_{2}+\mu_{i}}\kappa_{y}^{j_{2}}{y_{i}}^{2j_{2}+2\mu_{i}-1}}{\Gamma \left(j_{2}+\mu_{i}\right)j_{2}!}{\left(\frac{1+\kappa_{y}}{s_{i}}\right)}^{j_{2}+\mu_{i}}$$

It is clear that the κ-µ distribution is defined by two parameters, κ and µ. Here, parameter κ*_y_* is the Rician factor equal to the ratio of the dominant and scattered components, describing the CCI, and parameter *μ* is the number of clusters in the wireless environment; the CCI mean square values are denoted by *s_i_*, *i* = 1, 2, …, *L*. A modified Bessel function *I_v_*(*z*) is expanded to series by using ([18], Equation (2)).

The κ-µ is also general distribution: the κ-µ distribution becomes one-sided Gaussian distribution if µ = 0.5 and κ = 0; if µ = 1 and κ = 0, the κ-µ distribution becomes Rayleigh distribution; when µ = 1 and κ = *K*, the κ-µ distribution is Rician distribution, with *K* representing Rician *K* parameter; and if µ = *m* and κ = 0, the κ-µ distribution becomes Nakagami-*m* distribution, where *m* represents Nakagami-*m* fading severity parameter and *m* ≥ 1/2 [3].

The SIR *z_i_* has the PDF defined in [21]:

(6) $$p_{z_{i}}\left(z_{i}\right)=\int_{0}^{\infty }p_{x_{i}}\left(z_{i}y_{i}\right)p_{y_{i}}\left(y_{i}\right)y_{i}dy_{i}.$$

For our case of disturbances, it is:

(7) $$\begin{array}{l} p_{z_{i}}\left(z_{i}\right)=\frac{2}{e^{m_{i}\kappa_{x_{i}}+\mu_{i}\kappa_{y_{i}}}}\\ \times \sum_{j_{1}=0}^{+\infty }\overset{+\infty }{\underset{j_{2}=0}{\sum }}\frac{{\kappa_{x}}^{j_{1}}\kappa_{y}^{j_{2}}{m_{i}}^{2j_{1}+m_{i}}{\mu_{i}}^{2j_{2}+\mu_{i}}{s_{i}}^{j_{1}+m_{i}}{\left(\Omega_{i}\left(1+\kappa_{y_{i}}\right)\right)}^{j_{2}+\mu_{i}}{z_{i}}^{2j_{1}+2m_{i}-1}\Gamma \left(j_{1}+j_{2}+\mu_{i}+m_{i}\right)}{j_{1}!j_{2}!\Gamma \left(j_{1}+m_{i}\right)\Gamma \left(j_{2}+\mu_{i}\right){\left(\mu_{i}\Omega_{i}\left(1+\kappa_{y}\right)+m_{i}s_{i}{z_{i}}^{2}\right)}^{j_{1}+j_{2}+\mu_{i}+m_{i}}} \end{array}$$

The PDF of the SIR *z* from SC receiver output is calculated by dint of formula [22]:

(8) $$p_{z_{i}}\left(z\right)=Lp_{z_{i}}\left(z_{i}\right){\left(F_{z_{i}}\left(z_{i}\right)\right)}^{L-1}.$$

By substitutions of (4) and (7) in above expression, the PDF of the output SIR *z* becomes:

(9) $$\begin{array}{l} p_{z_{i}}\left(z\right)=\frac{2L}{e^{\left(m_{i}\kappa_{x_{i}}+\mu_{i}\kappa_{y_{i}}\right)L}}\\ \times \sum_{j_{1}=0}^{+\infty }\overset{+\infty }{\underset{j_{2}=0}{\sum }}\frac{{\kappa_{x}}^{j_{1}}\kappa_{y}^{j_{2}}{m_{i}}^{2j_{1}+m_{i}}{\mu_{i}}^{2j_{2}+\mu_{i}}{\left(1+\kappa_{y_{i}}\right)}^{j_{2}+\mu_{i}}{z_{i}}^{2j_{1}+2m_{i}-1}\Gamma \left(j_{1}+j_{2}+\mu_{i}+m_{i}\right)}{j_{1}!j_{2}!\Gamma \left(j_{1}+m_{i}\right)\Gamma \left(j_{2}+\mu_{i}\right){\left(\mu_{i}\left(1+\kappa_{y}\right)\left(\Omega_{i}/s_{i}\right)+m_{i}{z_{i}}^{2}\right)}^{j_{1}+j_{2}+\mu_{i}+m_{i}}}{\left(\frac{\Omega_{i}}{s_{i}}\right)}^{j_{2}+\mu_{i}}\\ \times (\sum_{j_{3}=0}^{+\infty }\overset{+\infty }{\underset{j_{4}=0}{\sum }}\frac{{\left(\kappa_{x}m_{i}\right)}^{j_{3}}{\left(\kappa_{y}\mu_{i}\right)}^{j_{4}}\Gamma \left(j_{3}+j_{4}+\mu_{i}+m_{i}\right)}{j_{3}!j_{4}!\Gamma \left(j_{3}+m_{i}\right)\Gamma \left(j_{4}+\mu_{i}\right)}{B_{\frac{m_{i}z^{2}}{\mu_{i}\left(1+\kappa_{y}\right)\left(\frac{\Omega_{i}}{s_{i}}\right)+m_{i}z^{2}}}\left(j_{3}+m_{i},j_{4}+\mu_{i}\right))}^{L-1} \end{array}.$$

The incomplete Beta function from the previous expression is represented by ([20], Equation (8.391)), as in [23] through Equations (6) and (7).

Based on these formulas, the PDF of SIR *z* is:

(10) $$\begin{array}{l} p_{z_{i}}\left(z\right)=\frac{2L}{e^{\left(m_{i}\kappa_{x_{i}}+\mu_{i}\kappa_{y_{i}}\right)L}}\sum_{j_{1}=0}^{+\infty }\overset{+\infty }{\underset{j_{2}=0}{\sum }}\frac{{\kappa_{x}}^{j_{1}}\kappa_{y}^{j_{2}}{m_{i}}^{2j_{1}+m_{i}}{\mu_{i}}^{2j_{2}+\mu_{i}}{\left(1+\kappa_{y_{i}}\right)}^{j_{2}+\mu_{i}}{z_{i}}^{2j_{1}+2m_{i}-1}\Gamma \left(j_{1}+j_{2}+\mu_{i}+m_{i}\right)}{j_{1}!j_{2}!\Gamma \left(j_{1}+m_{i}\right)\Gamma \left(j_{2}+\mu_{i}\right){\left(\mu_{i}\left(1+\kappa_{y}\right)\left(\Omega_{i}/s_{i}\right)+m_{i}{z_{i}}^{2}\right)}^{j_{1}+j_{2}+\mu_{i}+m_{i}}}{\left(\frac{\Omega_{i}}{s_{i}}\right)}^{j_{2}+\mu_{i}}\\ \times (\sum_{j_{3}=0}^{+\infty }\overset{+\infty }{\underset{j_{4}=0}{\sum }}\sum_{j_{5}=0}^{+\infty }\frac{{\left(\kappa_{x}m_{i}\right)}^{j_{3}}{\left(\kappa_{y}\mu_{i}\right)}^{j_{4}}\Gamma \left(j_{3}+j_{4}+\mu_{i}+m_{i}\right){\left(j_{3}+m_{i}\right)}_{j_{5}}{\left(1-j_{4}-\mu_{i}\right)}_{j_{5}}}{j_{3}!j_{4}!j_{5}!\Gamma \left(j_{3}+m_{i}\right)\Gamma \left(j_{4}+\mu_{i}\right)\left(j_{3}+m_{i}\right){\left(j_{3}+m_{i}+1\right)}_{j_{5}}}{{\left(\frac{m_{i}z^{2}}{\mu_{i}\left(1+\kappa_{y}\right)\left(\frac{\Omega_{i}}{s_{i}}\right)+m_{i}z^{2}}\right)}^{j_{3}+j_{5}+m_{i}})}^{L-1} \end{array}$$

**Figure 2:**
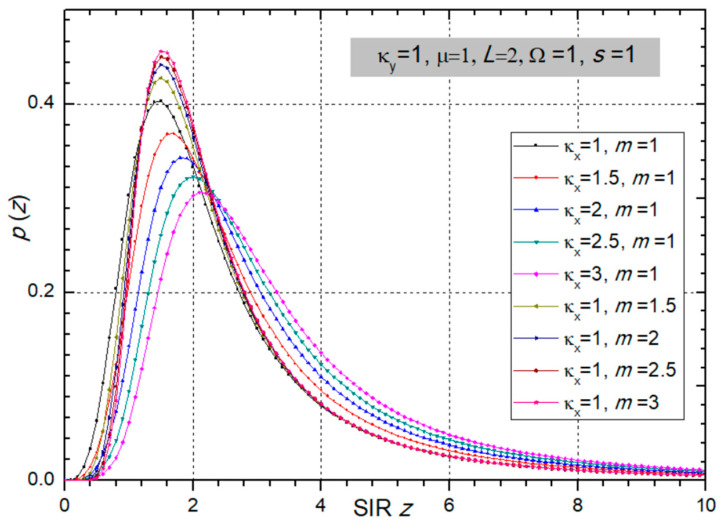
PDF of SIR *z* at the multi-branch SC receiver output for different values of fading parameters *m* and κ_x_. Other parameters are: κ_y_ = 1, µ = 1, *L* = 2, Ω = 1, and *s* = 1.

**Figure 3:**
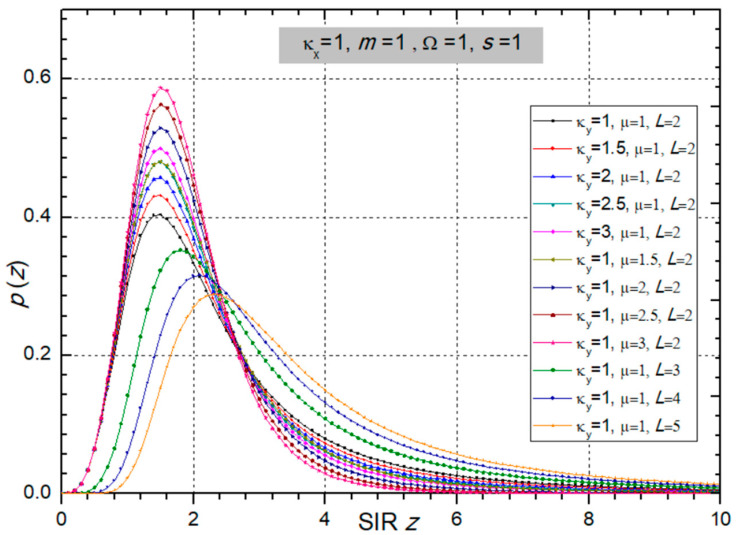
PDF versus SIR *z* at the multi-branch SC receiver output for variable CCI parameters κ_y_ and µ, and number of branches *L*. Other parameters are: κ_x_ = 1, *m* = 1, Ω = 1, and *s* = 1.

In the next two figures (Figure 2 and Figure 3), the PDF is presented versus SIR *z* for different values of fading and CCI parameters, powers, and number of input branches at the SC receiver. 

### 2.2. The Outage Probability of the Output SIR

Then, the CDF is given by dint of [21]:

(11) $$F_{z_{i}}\left(z_{i}\right)=\int_{0}^{z_{i}}p_{z_{i}}\left(t\right)dt$$

After replacement, the CDF of SIR *z_i_* is:

(12) $$\begin{array}{l} F_{z_{i}}\left(z_{i}\right)=\frac{2}{e^{m_{i}\kappa_{x_{i}}+\mu_{i}\kappa_{y_{i}}}}\\ \times \sum_{j_{1}=0}^{+\infty }\overset{+\infty }{\underset{j_{2}=0}{\sum }}\frac{{\kappa_{x}}^{j_{1}}\kappa_{y}^{j_{2}}{m_{i}}^{2j_{1}+m_{i}}{\mu_{i}}^{2j_{2}+\mu_{i}}{s_{i}}^{j_{1}+m_{i}}{\left(\Omega_{i}\left(1+\kappa_{y_{i}}\right)\right)}^{j_{2}+\mu_{i}}\Gamma \left(j_{1}+j_{2}+\mu_{i}+m_{i}\right)}{j_{1}!j_{2}!\Gamma \left(j_{1}+m_{i}\right)\Gamma \left(j_{2}+\mu_{i}\right)}\\ \times \int_{0}^{z_{i}}\frac{{z_{i}}^{2j_{1}+2m_{i}-1}}{{\left(\mu_{i}\Omega_{i}\left(1+\kappa_{y}\right)+m_{i}s_{i}{z_{i}}^{2}\right)}^{j_{1}+j_{2}+\mu_{i}+m_{i}}}dt \end{array}.$$

Using the incomplete Beta function *B_z_*(*a*, *b*) ([20], Formula (8.38)) to solve the integral in expression (12), we obtain the CDF of SIR *z_i_* as:

(13) $$\begin{array}{l} F_{z_{i}}\left(z_{i}\right)=\frac{1}{e^{m_{i}\kappa_{x_{i}}+\mu_{i}\kappa_{y_{i}}}}\sum_{j_{1}=0}^{+\infty }\overset{+\infty }{\underset{j_{2}=0}{\sum }}\frac{{\left(\kappa_{x}m_{i}\right)}^{j_{1}}{\left(\kappa_{y}\mu_{i}\right)}^{j_{2}}\Gamma \left(j_{1}+j_{2}+\mu_{i}+m_{i}\right)}{j_{1}!j_{2}!\Gamma \left(j_{1}+m_{i}\right)\Gamma \left(j_{2}+\mu_{i}\right)}\\ \times B_{\frac{m_{i}s_{i}z^{2}}{\mu_{i}\Omega_{i}\left(1+\kappa_{y}\right)+m_{i}s_{i}z^{2}}}\left(j_{1}+m_{i},j_{2}+\mu_{i}\right) \end{array}.$$

The Pout is defined as the probability that the instantaneous error probability exceeds a defined value or, equivalently, probability that the output SIR falls below a certain specified threshold. Mathematically [1], the Pout presents the CDF of the SIR *z* at the multi-branch SC receiver output ([21], Equation (6.82)):

(14) $$P_{out}\left(z\right)=F_{z}\left(z\right)={\left(F_{z_{i}}\left(z_{i}\right)\right)}^{L}.$$

For our system model that is also treated in [22], the final exact expression for Pout is:

(15) $$\begin{array}{l} P_{out}\left(z\right)=(\frac{1}{e^{m_{i}\kappa_{x}+\mu_{i}\kappa_{y}}}\sum_{j_{1}=0}^{+\infty }\overset{+\infty }{\underset{j_{2}=0}{\sum }}\frac{{\left(\kappa_{x}m_{i}\right)}^{j_{1}}{\left(\kappa_{y}\mu_{i}\right)}^{j_{2}}}{j_{1}!j_{2}!}\\ \times \frac{\Gamma \left(j_{1}+j_{2}+\mu_{i}+m_{i}\right)}{\Gamma \left(j_{1}+m_{i}\right)\Gamma \left(j_{2}+\mu_{i}\right)}{B_{\frac{m_{i}s_{i}{z_{i}}^{2}}{\mu_{i}\Omega_{i}\left(1+\kappa_{y}\right)+m_{i}s_{i}{z_{i}}^{2}}}\left(j_{1}+m_{i},j_{2}+\mu_{i}\right))}^{L} \end{array}.$$

Here, we provided an improved formula for Pout since the BX fading parameter λ is given by (3), and parameters κ for fading and CCI are taken to be different, namely κ_x_ ≠ κ_y_. This performance is displayed through the infinite series, which requires a reasonable number of terms to be summed to obtain fast convergence.

Again, we point out that BX is a general distribution, and the results obtained here can be compared with the results of earlier published papers [23,24,25] by setting the particular parameters values defined above. The obtained result in (15) is reduced to special cases of existing fading channels shown in earlier works.

The case of Pout in the presence of BX fading and Nakagami-*m* CCI is obtained by replacing the values of the CCI parameters to be κ*_y_* = 0 and µ = *m* > 0.5. In this case, we obtain expression (9) from [24]. The following published case in [25] can be obtained by replacing the CCI parameters to be κ*_y_* = *K* and µ = 1, when expression (15) will become expression (7) from [25], i.e., we obtain the Pout for the channel disturbed by BX fading and the Rician CCI which was published in [23]. Also, the case analyzed in [23,25] for Pout in the presence of Rician fading and Nakagami-*m* CCI will be obtained from (15) by setting the parameter *m* to be 1, κ*_y_* to be 0, and µ = *m*.

To observe the influence of the parameters of fading and CCI on the Pout, we plotted two figures with several graphs using Wolfram Mathematica and Origin. The correlation between *L* input branches in the SC receiver is assumed as minimal. Figures were created using Wolfram Mathematica and Origin. We took the next values for the following parameters: *m_i_* = *m*, µ*_i_* = µ, Ω*_i_* = Ω, *s_i_* = *s*.

**Figure 4:**
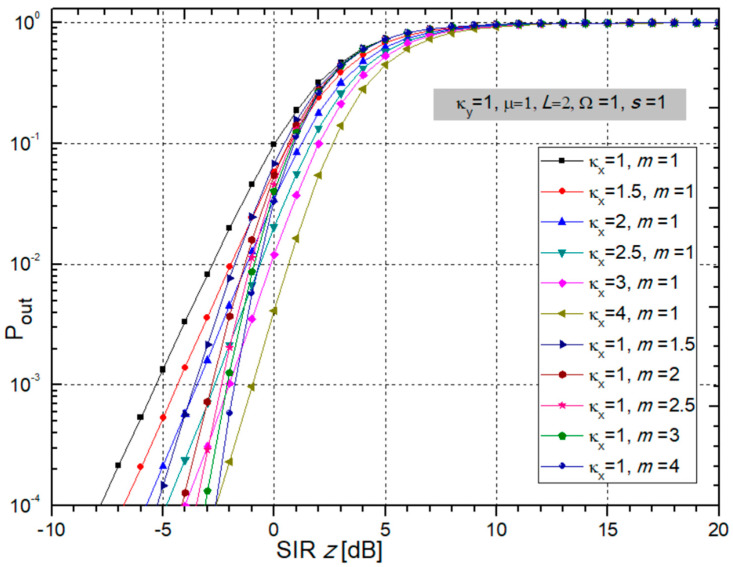
Outage probability of multi-branch SC receiver depending on SIR versus different values of fading parameters κ*_x_* and *m*.

From Figure 4, one can see that Pout increases when κ_x_ decreases. The Pout decreases as the fading parameter *m* increases, and system performance is better.

**Figure 5:**
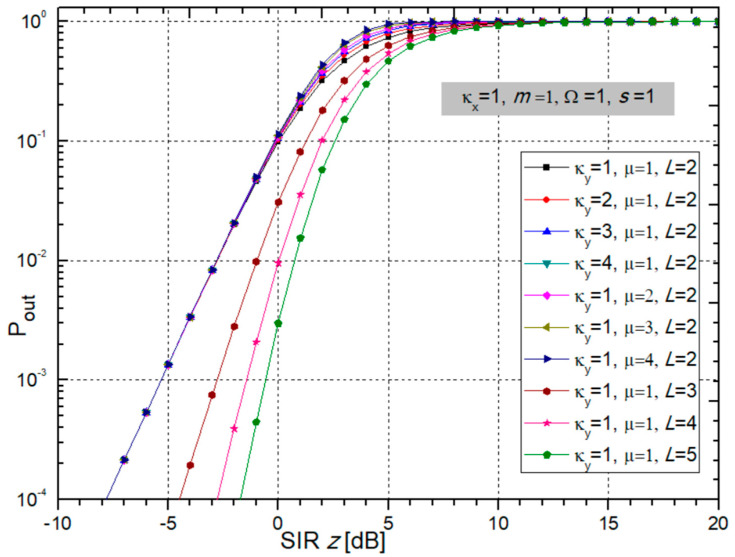
Pout of multi-branch SC receiver versus SIR considering different values of CCI parameters κ_y_ and µ, and number of branches *L*.

It can be seen from Figure 5 that Pout does not change significantly when changing the parameters κ*_y_* and µ. A larger value of the number of input diversity branches in the SC receiver improves the system performance by decreasing the Pout. This impact is also presented in Figure 5. It can be noticed that the maximum benefit is obtained when *L* changes from 1 to 2, and then the gain decreases.

Below the figures, we show tables with the number of required additions to achieve accuracy to the 5th decimal place in the series which present the Pout in (15). From Table 1, one can notice how it is necessary to sum a maximum of 16 terms to achieve that accuracy for the corresponding values of the system parameters.

It is visible from Table 1 and Table 2 that for all values of BX fading parameters and κ-µ CCI parameters, for all *z*, the number of additions does not exceed 9. One can notice from Table 2 that for *z* = −10 dB it is only 5, while for *z* = 0 dB and *z* = 10 dB it is slightly higher and increases to 9, depending on other parameters values.

### 2.3. The Channel Capacity

Channel capacity has great importance as a system performance measure of wireless systems. The CC is defined as the maximal rate at which information can be transmitted through a wireless channel with arbitrarily small BEP, without delay or complexity limits [26]:

(16) $$\frac{CC}{B}=\frac{1}{\ln\left(2\right)}\int_{0}^{\infty }\ln\left(1+z\right)p_{z}\left(z\right)dz$$

where *CC* is a label for Shannon capacity expressed in bits/s, and *B* marks transmission bandwidth expressed in Hz.

Deriving the expression of the normalized CC for such conditions, given by (16), is started from expressing the logarithmic function in the form [27]:

(17) $$\ln\left(1+x\right)=\sum_{i=0}^{+\infty }{\left(-1\right)}^{i}\frac{x^{i+1}}{\left(i+1\right)!}.$$

Using (10) and (17) in (16) gives the final form of the normalized CC:

(18) $$\begin{array}{l} \frac{CC}{B}=\frac{2L}{\ln\left(2\right)e^{\left(m_{i}\kappa_{x_{i}}+\mu_{i}\kappa_{y_{i}}\right)L}}\sum_{j_{1}=0}^{+\infty }\sum_{j_{2}=0}^{+\infty }\overset{+\infty }{\underset{j_{3}=0}{\sum }}\frac{{\left(-1\right)}^{j_{1}}{\kappa_{x}}^{j_{2}}\kappa_{y}^{j_{3}}{m_{i}}^{2j_{2}+Lm_{i}}{\mu_{i}}^{j_{3}}\Gamma \left(j_{2}+j_{3}+\mu_{i}+m_{i}\right)}{\left(j_{1}+1\right)!j_{2}!j_{3}!\Gamma \left(j_{2}+m_{i}\right)\Gamma \left(j_{3}+\mu_{i}\right)}{\left(\frac{s_{i}}{\mu_{i}\Omega_{i}\left(1+\kappa_{y}\right)}\right)}^{j_{2}+Lm_{i}}\\ \times (\sum_{j_{4}=0}^{+\infty }\overset{+\infty }{\underset{j_{5}=0}{\sum }}\sum_{j_{6}=0}^{+\infty }\frac{{\left(\kappa_{x}m_{i}\right)}^{j_{4}}{\left(\kappa_{y}\mu_{i}\right)}^{j_{5}}\Gamma \left(j_{4}+j_{5}+\mu_{i}+m_{i}\right){\left(j_{4}+m_{i}\right)}_{j_{6}}{\left(1-j_{5}-\mu_{i}\right)}_{j_{6}}}{j_{4}!j_{5}!j_{6}!\Gamma \left(j_{4}+m_{i}\right)\Gamma \left(j_{5}+\mu_{i}\right){\left(j_{4}+m_{i}+1\right)}_{j_{6}}\left(j_{4}+m_{i}\right)}{{\left(\frac{m_{i}s_{i}}{\mu_{i}\Omega_{i}\left(1+\kappa_{y}\right)}\right)}^{j_{4}+j_{6}})}^{L-1}\\ \times \int_{0}^{\infty }\frac{{z_{i}}^{j_{1}+2j_{2}+2Lj_{4}-j_{4}+2Lj_{6}-j_{6}+2Lm_{i}+m_{i}}}{{\left(1+\frac{m_{i}s_{i}}{\mu_{i}\Omega_{i}\left(1+\kappa_{y}\right)}{z_{i}}^{2}\right)}^{j_{2}+j_{3}+Lj_{4}-j_{4}+Lj_{6}-j_{6}+Lm_{i}+\mu_{i}}}dz \end{array}.$$

Presenting integral from (18) using form ([20], Formula 3.251):

(19) $$\int_{0}^{\infty }\frac{x^{\mu -1}}{{\left(1+\beta x^{p}\right)}^{v}}dx=\frac{1}{p}\beta^{-\frac{\mu }{p}}B\left(\frac{\mu }{p},v-\frac{\mu }{p}\right),$$

we obtain:

(20) $$\begin{array}{l} \frac{CC}{B}=\frac{L}{\ln\left(2\right)e^{\left(m_{i}\kappa_{x_{i}}+\mu_{i}\kappa_{y_{i}}\right)L}}(\sum_{j_{1}=0}^{+\infty }\sum_{j_{2}=0}^{+\infty }\overset{+\infty }{\underset{j_{3}=0}{\sum }}\frac{{\left(-1\right)}^{j_{1}}{\left(\kappa_{x}m_{i}\right)}^{j_{2}}{\left(\kappa_{y}\mu_{i}\right)}^{j_{3}}\Gamma \left(j_{2}+j_{3}+\mu_{i}+m_{i}\right)}{\left(j_{1}+1\right)!j_{2}!j_{3}!\Gamma \left(j_{2}+m_{i}\right)\Gamma \left(j_{3}+\mu_{i}\right)}\\ \times (\sum_{j_{4}=0}^{+\infty }\overset{+\infty }{\underset{j_{5}=0}{\sum }}\sum_{j_{6}=0}^{+\infty }{\left(\frac{{\left(\kappa_{x}m_{i}\right)}^{j_{4}}{\left(\kappa_{y}\mu_{i}\right)}^{j_{5}}\Gamma \left(j_{4}+j_{5}+\mu_{i}+m_{i}\right){\left(j_{4}+m_{i}\right)}_{j_{6}}{\left(1-j_{5}-\mu_{i}\right)}_{j_{6}}}{j_{4}!j_{5}!j_{6}!\Gamma \left(j_{4}+m_{i}\right)\Gamma \left(j_{5}+\mu_{i}\right)\left(j_{4}+m_{i}\right){\left(j_{4}+m_{i}+1\right)}_{j_{6}}}\right)}^{L-1}\\ \times {\left(\frac{\mu_{i}\left(1+\kappa_{y}\right)}{m_{i}}\frac{\Omega_{i}}{s_{i}}\right)}^{\frac{j_{1}+j_{4}+j_{6}+m_{i}+1}{2}}B\left(\frac{j_{1}+2j_{2}+\left(j_{4}+j_{6}\right)\left(2L-1\right)+m_{i}\left(2L+1\right)+1}{2},\frac{2j_{3}-j_{1}-j_{4}-j_{6}+2\mu_{i}-m_{i}-1}{2}\right)) \end{array}$$

A graphical presentation of the normalized CC, i.e., CC/B, at the output of the multi-branch SC combiner is given in the next two figures versus fading and CCI powers ratio *w_i_* = Ω*_i_*/*s_i_*.

**Figure 6:**
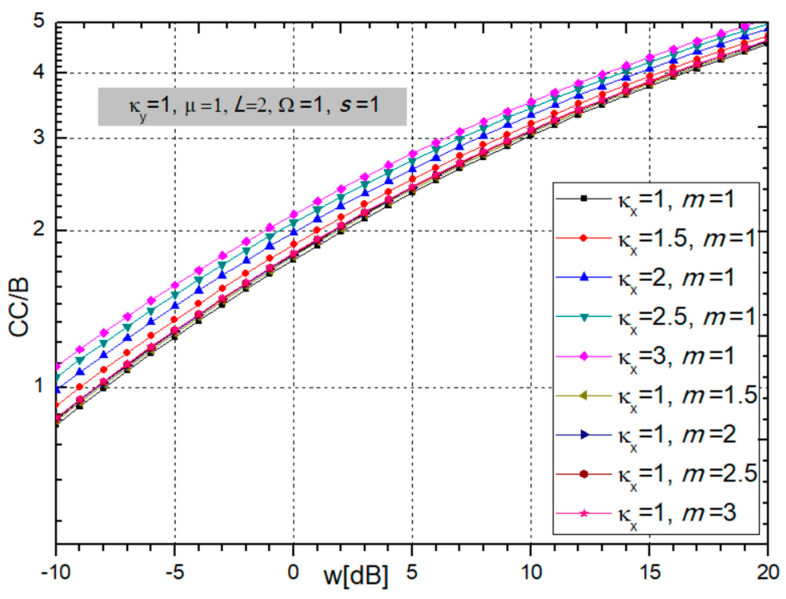
Normalized channel capacity for different values of BX fading parameters κ_x_ and *m*.

Figure 6 shows that an increase in the fading parameter κ_x_ increases the CC, while parameter *m* does not have an effect on the channel capacity.

**Figure 7:**
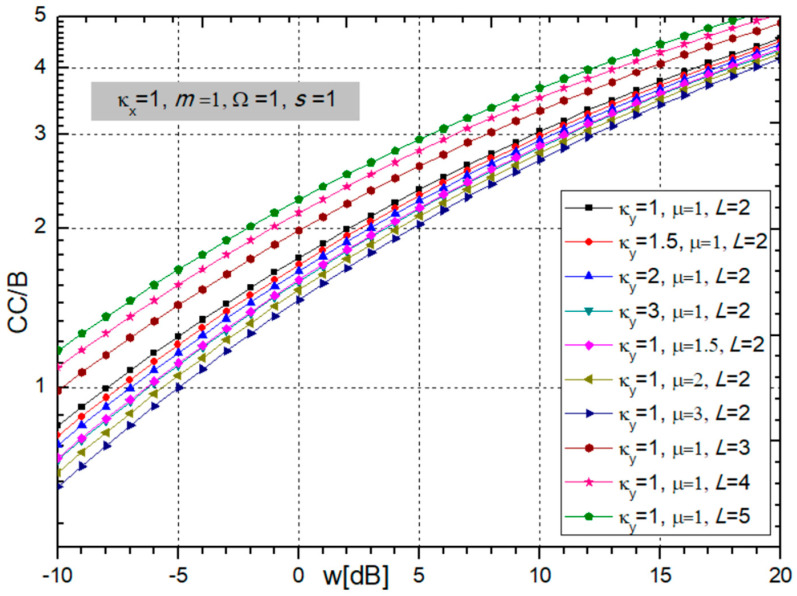
Normalized channel capacity for different values of CCI parameters κ_y_ and µ and number of branches *L*.

From Figure 7, it is obvious that the number of branches at the receiver input, *L.*, improves the magnitude of the channel capacity. It should be emphasized that the increase in capacity is greatest when diversity combining is introduced, that is, when *L* changes from 1 to 2, and after that, the increase decreases and further growth in the number of branches is no longer economically justified. Contrary to that, an increase in CCI parameters κ_y_ and µ worsens the system performance by reducing the channel capacity.

Afterwards, we present tables with the required number of terms in sums, in expression (20) for capacity, for achieving accuracy at the 5th decimal place.

Table 3 shows that when the parameters κ*_x_* and *m* increase, the number of elements that need to be added in order to achieve convergence to the fifth decimal increases, and the series converges more slowly. It is necessary to add between 8 and 17 additions for all values of fading and CCI parameters, as well as powers.

As Table 4 shows, as parameters κ_y_ and µ grow, the number of elements that need to be added in order to achieve convergence to the fifth decimal increases, and the series does not converge as quickly. When parameter *L* increases, the number of elements for convergence tends to 10 for all values of *w_i_* [dB]. This is also possible to see from Table 4.

### 2.4. The Moment-Generating Function

In this subsection, we will derive the MGF for the wireless system model from Figure 1 in the presence of BX fading and κ-µ CCI. The MGF is an important statistical function for each distribution with many advantages, as is analysis of sums of Random Variables (RVs). The MGF of RV defines all moments of the RV, which determines the name—moment-generating function. It is important that the MGF determines the distribution uniquely, if it exists. Consequently, two RVs have the same distribution if they have the same MGF. So, the distribution is determined after finding the MGF, especially important in the case of a complicated PDF.

As the parameters κ_y_ and µ increase, the number of elements that need to be added in order to achieve convergence to the fifth decimal increases, and the series does not converge as quickly. When parameter *L* increases, the number of elements for convergence tends to 10 for all values of *w_i_* [dB].

The formula for derivation of the MGF is ([28], Equation (6)):

(21) $$M_{z}\left(h\right)=\bar{e^{hz}}=\int_{0}^{\infty }e^{-hz}p_{z_{i}}\left(z\right)dz.$$

Using (10) for the PDF of SIR *z* in Formula (21) for MGF, we obtain:

(22) $$\begin{array}{l} M_{z}\left(h\right)=\frac{2L}{e^{\left(m_{i}\kappa_{x_{i}}+\mu_{i}\kappa_{y_{i}}\right)L}}\sum_{j_{1}=0}^{+\infty }\overset{+\infty }{\underset{j_{2}=0}{\sum }}\frac{{\kappa_{x}}^{j_{1}}\kappa_{y}^{j_{2}}{m_{i}}^{j_{1}-j_{2}-\mu_{i}}{\mu_{i}}^{2j_{2}+\mu_{i}}{\left(1+\kappa_{y_{i}}\right)}^{j_{2}+\mu_{i}}\Gamma \left(j_{1}+j_{2}+\mu_{i}+m_{i}\right)}{j_{1}!j_{2}!\Gamma \left(j_{1}+m_{i}\right)\Gamma \left(j_{2}+\mu_{i}\right)}{\left(\frac{\Omega_{i}}{s_{i}}\right)}^{j_{2}+\mu_{i}}\\ \times (\frac{1}{e^{m_{i}\kappa_{x_{i}}+\mu_{i}\kappa_{y_{i}}}}{\sum_{j_{3}=0}^{+\infty }\overset{+\infty }{\underset{j_{4}=0}{\sum }}\sum_{j_{5}=0}^{+\infty }\frac{{\left(\kappa_{x}m_{i}\right)}^{j_{3}}{\left(\kappa_{y}\mu_{i}\right)}^{j_{4}}{\left(j_{3}+m_{i}\right)}_{j_{5}}{\left(1-j_{4}-\mu_{i}\right)}_{j_{5}}\Gamma \left(j_{3}+j_{4}+\mu_{i}+m_{i}\right)}{j_{3}!j_{4}!j_{5}!\Gamma \left(j_{3}+m_{i}\right)\Gamma \left(j_{4}+\mu_{i}\right)\left(j_{3}+m_{i}\right){\left(j_{3}+m_{i}+1\right)}_{j_{5}}})}^{L-1}\\ \times \int_{0}^{\infty }\frac{{z_{i}}^{2j_{1}+2Lj_{3}-2j_{3}+2Lj_{5}-2j_{5}+2Lm_{i}-1}}{{\left({\left(\sqrt{\frac{\mu_{i}\Omega_{i}\left(1+\kappa_{y}\right)}{m_{i}s_{i}}}\right)}^{2}+{z_{i}}^{2}\right)}^{1-\left(j_{3}+j_{5}-j_{1}-j_{2}-Lj_{3}-Lj_{5}-Lm_{i}-\mu_{i}+1\right)}}e^{-hz}dz \end{array}.$$

If the development ([20], Formula (3.389)):

(23) $$\int_{0}^{\infty }\frac{x^{2v-1}e^{-\mu x}}{{\left(u^{2}+x^{2}\right)}^{1-q}}dx=\frac{u^{2v+2q-2}}{2\sqrt{\pi }\Gamma \left(1-q\right)}G_{13}^{31}\left(\frac{\mu^{2}u^{2}}{4}|\begin{array}{l} 1-v\\ 1-q-v,0,\frac{1}{2} \end{array}\right),$$

would be introduced into (22), the MGF for output SIR *z* becomes:

(24) $$\begin{array}{l} M_{z}\left(h\right)=\frac{L}{\sqrt{\pi }e^{\left(m_{i}\kappa_{x_{i}}+\mu_{i}\kappa_{y_{i}}\right)L}}\sum_{j_{1}=0}^{+\infty }\overset{+\infty }{\underset{j_{2}=0}{\sum }}\frac{{\kappa_{x}}^{j_{1}}\kappa_{y}^{j_{2}}{m_{i}}^{j_{1}+1}{\mu_{i}}^{j_{2}-1}{\left(1+\kappa_{y_{i}}\right)}^{j_{2}+\mu_{i}}\Gamma \left(j_{1}+j_{2}+\mu_{i}+m_{i}\right)}{j_{1}!j_{2}!\Gamma \left(j_{1}+m_{i}\right)\Gamma \left(j_{2}+\mu_{i}\right){\left(1+\kappa_{y}\right)}^{j_{2}+\mu_{i}+1}}\left(\frac{s_{i}}{\Omega_{i}}\right)\\ \times {\left(\sum_{j_{3}=0}^{+\infty }\overset{+\infty }{\underset{j_{4}=0}{\sum }}\sum_{j_{5}=0}^{+\infty }\frac{{\left(\kappa_{x}m_{i}\right)}^{j_{3}}{\left(\kappa_{y}\mu_{i}\right)}^{j_{4}}{\left(j_{3}+m_{i}\right)}_{j_{5}}{\left(1-j_{4}-\mu_{i}\right)}_{j_{5}}\Gamma \left(j_{3}+j_{4}+\mu_{i}+m_{i}\right)}{j_{3}!j_{4}!j_{5}!\Gamma \left(j_{3}+m_{i}\right)\Gamma \left(j_{4}+\mu_{i}\right)\left(j_{3}+m_{i}\right){\left(j_{3}+m_{i}+1\right)}_{j_{5}}}\right)}^{L-1}\\ \times \frac{1}{\Gamma \left(j_{1}+j_{2}+Lj_{3}-j_{3}+Lj_{5}-j_{5}+Lm_{i}+\mu_{i}\right)}G_{13}^{31}\left(\frac{h^{2}\mu_{i}\left(1+\kappa_{y}\right)}{4m_{i}}\left(\frac{\Omega_{i}}{s_{i}}\right)|\begin{array}{l} j_{3}-j_{1}-Lj_{3}+j_{5}-Lj_{5}-Lm_{i}+1\\ j_{2}+\mu_{i},0,\frac{1}{2} \end{array}\right) \end{array}.$$

Here, G[·] means the Meijer’s G-function [29].

### 2.5. The ABEP for Binary Frequency Shift Keying Modulation

The ABEP is a very important system performance of the first order that best describes the behavior of characteristics of a wireless system. Therefore, it is very important to determine ABEP in the most efficient way possible. For efficient derivation of ABEP, the MGF is used when we do not know PDF reliably.

First, we evaluate the ABEP based on the MGF for BFSK modulation without numerical integrations. The formula is [1]:

(25) $$P_{\mathrm{be}}(\Omega_{0})=0{.5M}_{z}(0.5).$$

Finally, the ABEP for non-coherent BFSK modulation is obtained by substituting (24) into (25) and presented in the next figures.

**Figure 8:**
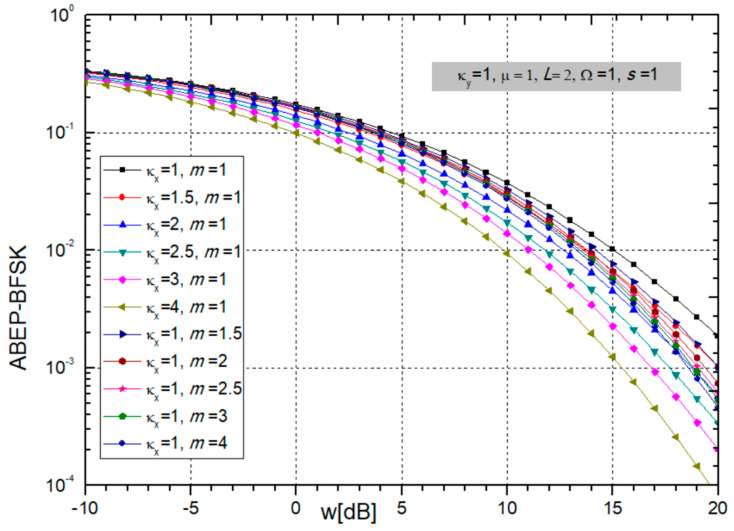
ABEP for BFSK modulation: parameters κ_x_ and *m* are changing, and constant parameters are κ_y_ = 1, µ = 1, *L* = 2, Ω = 1, *s* = 1.

**Figure 9:**
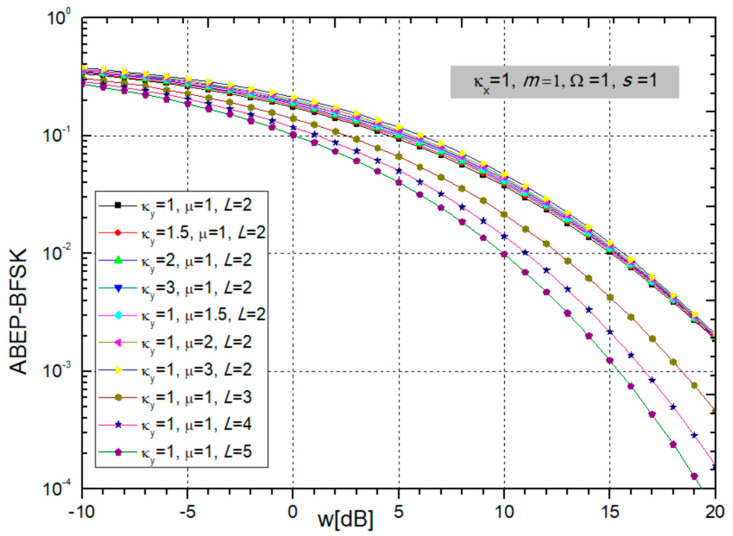
ABEP for BFSK modulation: changeable CCI parameters κ_y_ and µ, and number of branches *L*; and constant are κ_x_ = 1, *m* = 1, Ω = 1, *s* = 1.

It is possible to observe from Figure 8 and Figure 9 that ABEP decreases with an increase in BX fading parameters κ_x_ and *m*, power ratio *w_i_,* and the number of SC combiner input branches *L*. In such a case, system performance is improved. Evidently, the ABER decreases with increasing *L*, but not linearly. The maximum benefit is when *L* increases from 1 to 2 and then decreases with further growth of *L*. On the other hand, CCI parameters κ_y_ and µ do not make much of an impact on the ABEP.

After the figures, we show tables illustrating the required number of additions in the sum in expression (25) to achieve accuracy to the 5th significant digit.

It is visible from Table 5 that when the parameters κ_x_ and *m* increase, the number of elements that need to be added in order to achieve convergence to the fifth decimal increases, and the series does not converge so quickly.

As can be seen in Table 6, as the parameters κ_y_ and µ increase, the number of additions necessary to achieve faster convergence increases, and the series converges more slowly. When *L* is increasing, the number of elements tends to be smaller, so for *z* = −10 dB and *z* = 0 dB, it is 8, and for *z* = 10 dB, the number of elements is 7.

### 2.6. The ABEP for Binary Differential Phase-Shift Keying Modulation

We evaluate here the MGF-based ABEP for BDPSK modulation. The formula is given by [1]:

(26) $$P_{\mathrm{be}}(\Omega_{0})=0{.5M}_{z}\left(1\right),\mathrm{for}\mathrm{BDPSK}.$$

Based on replacing (24) to form (26), the ABEP for BDPSK modulation is obtained and presented in the next figures versus *w_i_* = Ω_i_/*s_i_* for different sets of parameters.

**Figure 10:**
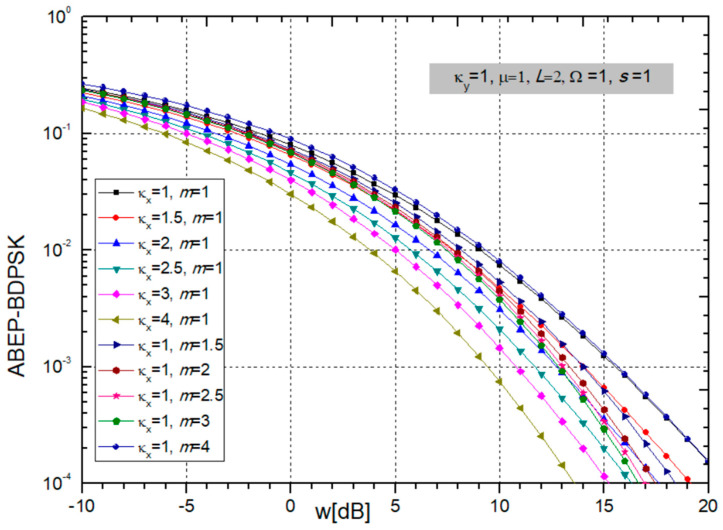
ABEP for BDPSK modulation when parameters κ_x_ and *m* are changing. Other parameters values are constant: κ_y_ = 1, µ = 1, *L* = 2, and powers: Ω = 1, *s* = 1.

In the case of ABEP for BDPSK modulation presented in Figure 10, it can be noticed that ABEP decreases with increasing fading parameter κ_x_, and power ratio *w_i_,* but ABEP is bigger for larger values of parameter *m*, and the system performance deteriorates.

**Figure 11:**
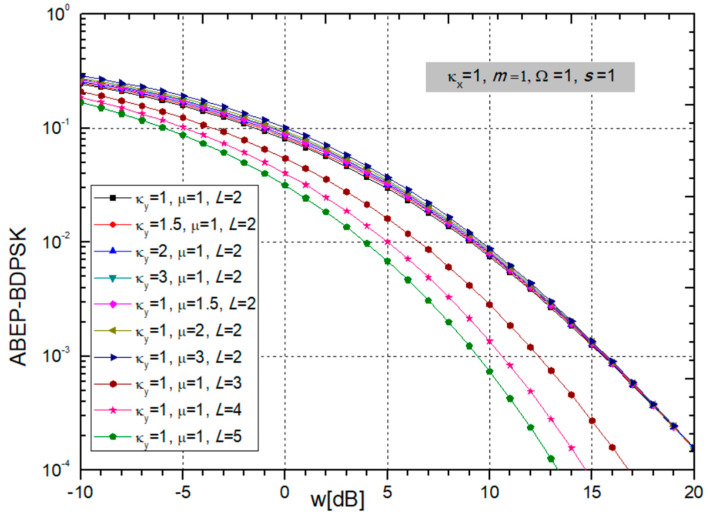
MGF-based ABEP for BDPSK modulation: CCI parameters κ_y_ and µ are varying, and number of branches *L,* while constant are fading parameters κ_x_ = 1, *m* = 1, and powers Ω = 1, *s* = 1.

In the situation in Figure 11, the influence of CCI parameters κ_y_ and µ and the number of input branches *L* are shown. When *L* is increasing, the ABEP is becoming smaller, thereby improving system performance. The decrease in ABEP is greatest when L increases from 1 to 2, then somewhat less from 2 to 3, and so on. Therefore, to improve the performance, it is quite sufficient to take an SC combiner with 2 or 3 branches. On the other hand, parameters κ_y_ and µ do not significantly affect the size of the ABEP.

Afterwards, we provide two tables showing the number of additions required in the sum in expression (26) to achieve the required accuracy.

From Table 7, we see that as the parameters κ_x_ and *m* increase, the number of elements that need to be added in order to achieve convergence to the fifth decimal increases, and the series converges more slowly. It is necessary to have in sum between 8 and 14 additions for all values of parameters participating in expressions.

When the parameters κ_y_ and µ increase, as shown in Table 8, the number of additions in the sum required to achieve convergence to the fifth decimal increases, and the series converges more slowly. When *L* is bigger than 2, the number of elements decreases significantly, so for z = −10 dB it is 8, for z = 0 dB it is 7, and for z = 10 dB the number of elements is only 5 or 6, depending on other parameters values.

## 3. Second-Order System Performance

Between the second-order system performances, the most important are the level crossing rate and average fade duration. The LCR and AFD characterize the aspects of the dynamic temporal behavior of envelope fluctuations. Knowing these magnitudes can help us better understand and combat the disturbing effects of fading.

The LCR is the number of crossing the specified level in a positive (or negative) direction. The AFD shows the average time that the signal envelope spends below that specified threshold level.

### 3.1. Level Crossing Rate

The LCR of the SIR at the output of a multi-branch SC receiver is actually the mean value of the first derivative of the SIR at the receiver output. Accordingly, it is necessary to average the first derivative by an integration [30]:

(27) $$N_{z_{i}}\left(z_{i}\right)=\int_{0}^{\infty }{\dot{z}}_{i}p_{{\dot{z}}_{i}z_{i}}\left({\dot{z}}_{i}z_{i}\right)d{\dot{z}}_{i}$$

Since we need ${\dot{z}}_{i}$ (the first derivative of SIR z_i_) to obtain LCR, let us repeat the calculation from [31] with λ from (3):

(28) $${\dot{z}}_{i}=\frac{1}{y_{i}}{\dot{x}}_{i}-\frac{x_{i}}{y_{i}^{2}}{\dot{y}}_{i}.$$

The first derivatives of ${\dot{x}}_{i}$ and ${\dot{y}}_{i}$ are distributed by Gauss, subsequently ${\dot{z}}_{i}$ has Gaussian distribution with zero mean value:

(29) $${\bar{\dot{z}}}_{i}=\frac{1}{y_{i}}{\bar{\dot{x}}}_{i}-\frac{x_{i}}{y_{i}^{2}}{\bar{\dot{y}}}_{i}=0.$$

${\dot{z}}_{i}$ has the variance:

(30) $$\sigma_{{\dot{z}}_{i}}^{2}=\frac{1}{y_{i}^{2}}\sigma_{{\dot{x}}_{i}}^{2}+\frac{x_{i}^{2}}{y_{i}^{4}}\sigma_{{\dot{y}}_{i}}^{2}.$$

The variance of ${\dot{x}}_{i}$ is ([16], Equation (2.4)):

(31) $$\sigma_{{\dot{x}}_{i}}^{2}=\frac{\pi^{2}f_{m}^{2}\Omega_{i}}{m_{i}}.$$

CCI’s derivative ${\dot{y}}_{i}$ has the variance:

(32) $$\sigma_{{\dot{y}}_{i}}^{2}=\pi^{2}f_{m}^{2}\frac{s_{i}}{\mu_{i}\left(\kappa_{y}+1\right)}.$$

In expressions (31) and (32), Doppler frequency is marked with f_m_.

After substitution of the last two expressions in (30), the variance of ${\dot{z}}_{i}$ becomes:

(33) $$\sigma_{{\dot{z}}_{i}}^{2}=\frac{\pi^{2}f_{m}^{2}}{y_{i}^{2}}\left(\frac{\mu_{i}\Omega_{i}\left(\kappa_{y}+1\right)+m_{i}s_{i}z_{i}^{2}}{m_{i}\mu_{i}\left(\kappa_{y}+1\right)}\right).$$

Let us now determine the conditional PDFs (CPDF) of ${\dot{z}}_{i}$ and z_i_. They are [32]:

(34) $$p_{{\dot{z}}_{i}}\left({\dot{z}}_{i}|z_{i}y_{i}\right)=\frac{1}{\sqrt{2\pi }\sigma_{{\dot{z}}_{i}}}e^{-\frac{{\dot{z}}_{i}^{2}}{2\sigma_{{\dot{z}}_{i}}^{2}}},$$

(35) $$p_{z_{i}}\left(z_{i}|y_{i}\right)=\left|\frac{dx_{i}}{dz_{i}}\right|p_{x_{i}}\left(z_{i}y_{i}\right)=y_{i}p_{x_{i}}\left(z_{i}y_{i}\right).$$

Now, we should find the conditional joint PDF of z_i_, ${\dot{z}}_{i}$ and y_i_ [21]:

(36) $$p_{{\dot{z}}_{i}z_{i}y_{i}}\left({\dot{z}}_{i}z_{i}y_{i}\right)=p_{{\dot{z}}_{i}}\left({\dot{z}}_{i}|z_{i}y_{i}\right)p_{y_{i}}\left(y_{i}\right)y_{i}p_{x_{i}}\left(z_{i}y_{i}\right).$$

Then, the joint PDF of *z_i_* and ż_i_ is [21]:

(37) $$p_{{\dot{z}}_{i}z_{i}}\left({\dot{z}}_{i}z_{i}\right)=\int_{0}^{\infty }p_{{\dot{z}}_{i}z_{i}y_{i}}\left({\dot{z}}_{i}z_{i}y_{i}\right)dy_{i}.$$

Some replacements are performed in (27), and the LCR of SIR *z_i_* becomes:

(38) $$\begin{array}{l} N_{z_{i}}\left(z_{i}\right)=\frac{\sqrt{2\pi }f_{m}}{e^{m_{i}\kappa_{x_{i}}+\mu_{i}\kappa_{y_{i}}}}\sum_{j_{1}=0}^{+\infty }\overset{+\infty }{\underset{j_{2}=0}{\sum }}\frac{{\kappa_{x}}^{j_{1}}\kappa_{y}^{j_{2}}{m_{i}}^{2j_{1}+m_{i}-\frac{1}{2}}{\mu_{i}}^{2j_{2}+\mu_{i}-\frac{1}{2}}}{j_{1}!j_{2}!\Gamma \left(j_{1}+m_{i}\right)}\cdot \\ \times \frac{{\left(\Omega_{i}\left(1+\kappa_{y}\right)\right)}^{j_{2}+\mu_{i}-\frac{1}{2}}{s_{i}}^{j_{1}+m_{i}-\frac{1}{2}}{z_{i}}^{2j_{1}+2m_{i}-1}\Gamma \left(j_{1}+j_{2}+m_{i}+\mu_{i}-1/2\right)}{\Gamma \left(j_{2}+\mu_{i}\right){\left(\mu_{i}\Omega_{i}\left(1+\kappa_{y}\right)+m_{i}s_{i}{z_{i}}^{2}\right)}^{j_{1}+j_{2}+m_{i}+\mu_{i}-1}} \end{array}$$

The LCR of the output SIR *z* is calculated using formula ([33], Equation (8)):

(39) $$N_{z}\left(z\right)=L{\left(F_{z_{i}}\left(z_{i}\right)\right)}^{L-1}N_{z_{i}}\left(z_{i}\right).$$

Using expressions (39) and (13), we obtain the LCR at the output of the multi-branch SC receiver in the form:

(40) $$\begin{array}{l} N_{z}\left(z\right)=\frac{L\sqrt{2\pi }f_{m}}{e^{m_{i}\kappa_{x_{i}}+\mu_{i}\kappa_{y_{i}}}}\sum_{j_{1}=0}^{+\infty }\overset{+\infty }{\underset{j_{2}=0}{\sum }}\frac{{\kappa_{x}}^{j_{1}}\kappa_{y}^{j_{2}}{m_{i}}^{2j_{1}+m_{i}-\frac{1}{2}}{\mu_{i}}^{2j_{2}+\mu_{i}-\frac{1}{2}}{\left(\Omega_{i}\left(1+\kappa_{y}\right)\right)}^{j_{2}+\mu_{i}-\frac{1}{2}}}{j_{1}!j_{2}!\Gamma \left(j_{1}+m_{i}\right)}\\ \times \frac{{s_{i}}^{j_{1}+m_{i}-\frac{1}{2}}{z_{i}}^{2j_{1}+2m_{i}-1}\Gamma \left(j_{1}+j_{2}+m_{i}+\mu_{i}-1/2\right)}{\Gamma \left(j_{2}+\mu_{i}\right){\left(\mu_{i}\Omega_{i}\left(1+\kappa_{y}\right)+m_{i}s_{i}{z_{i}}^{2}\right)}^{j_{1}+j_{2}+m_{i}+\mu_{i}-1}}\\ \times (\frac{1}{e^{m_{i}\kappa_{x_{i}}+\mu_{i}\kappa_{y_{i}}}}\sum_{j_{3}=0}^{+\infty }\overset{+\infty }{\underset{j_{4}=0}{\sum }}\frac{{\left(\kappa_{x}m_{i}\right)}^{j_{3}}{\left(\kappa_{y}\mu_{i}\right)}^{j_{4}}\Gamma \left(j_{3}+j_{4}+\mu_{i}+m_{i}\right)}{j_{3}!j_{4}!\Gamma \left(j_{3}+m_{i}\right)\Gamma \left(j_{4}+\mu_{i}\right)}{B_{\frac{m_{i}s_{i}z^{2}}{\mu_{i}\Omega_{i}\left(1+\kappa_{y}\right)+m_{i}s_{i}z^{2}}}\left(j_{3}+m_{i},j_{4}+\mu_{i}\right))}^{L-1} \end{array}$$

This expression differs from ([31], Equation (22)) because the connection from (3) is used:

$$\lambda_{i}^{2}=\kappa_{x}\Omega_{i}$$

and κ*_x_* and κ*_y_* are different. With these improvements, we increased the generality of the expression.

This expression can be compared with the expression ([34], Equation (24)). The expression ([34], Equation (24)) will be obtained by setting the appropriate parameter values, as defined below the expressions for PDFs of BX fading and κ-µ CCI, and using λ from (3) and the same values for Rician factor of both, fading and CCI (κ*_x_* = κ*_y_* = *K*).

**Figure 12:**
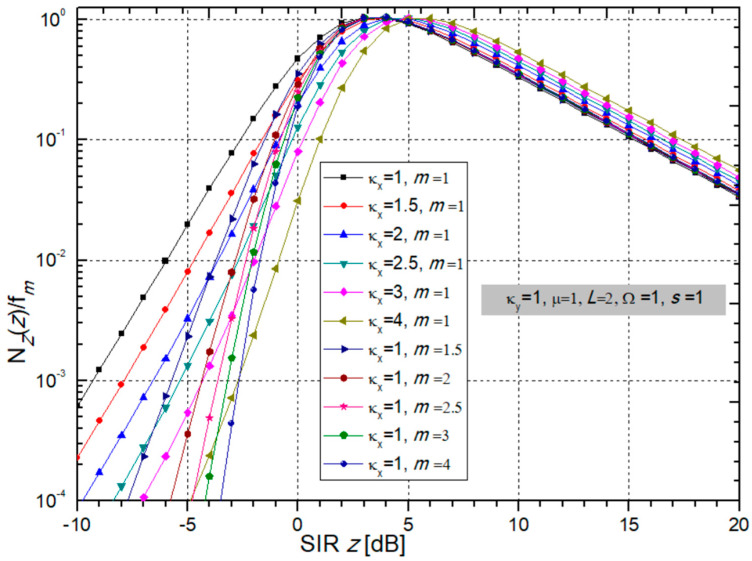
The LCR, normalized by Doppler frequency f_m_, versus output SIR for different sets of BX fading parameters κ_x_ and m; CCI parameters are: κ_y_ = 1 and µ = 1, and powers: Ω = 1, s = 1.

**Figure 13:**
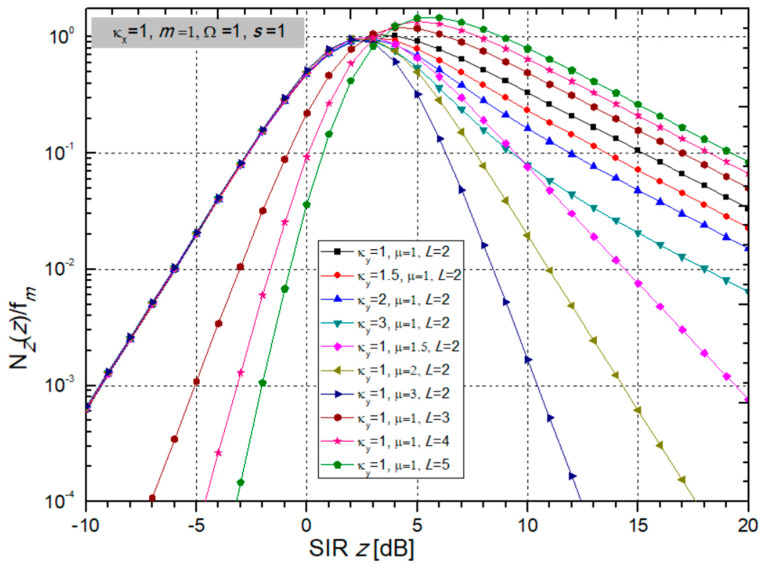
Normalized LCR depending on SIR with variable CCI parameters κ*_y_* and µ and number of branches *L*, while BX fading parameters remain constant: κ*_x_* = 1 and *m* = 1, as well as powers Ω = 1 and *s* = 1.

Now, a few graphical presentations of the normalized LCR at the output of the multi-branch SC receiver given by (40) are shown in Figure 12 and Figure 13 in order to examine the influence of BX fading parameters and κ-µ CCI parameters.

One can see from Figure 12 that due to the increase in BX fading parameters κ*_x_* and *m*, the LCR decreases for negative values of output SIR *z* (higher CCI), while for positive values of SIR, LCR decreases, and the system has better performance for all parameters.

In Figure 13, the normalized LCR is presented versus SIR for variable κ-µ CCI parameters κ*_y_* and µ, and number of receiver input branches *L*, while BX fading parameters and powers remain permanent values.

It is possible to notice that an increase in *L* improves performance because it reduces the LCR for all values of SIR *z*. When µ increases for positive *z*, the LCR drops, thereby improving the system performance. The influence of CCI parameter κ*_y_* is negligible for *z* < 0, while an increase in κ*_y_* causes a decrease in LCR for *z* > 0.

In the continuation are shown tables with the necessary number of additions in the sum in (40) to achieve fast convergence of those sums.

When the parameters κ*_x_* and *m* increase for *z* = −10 [dB], the number of elements that need to be added to achieve convergence to the fifth decimal is only 5. For *z* = 0 dB and *z* = 10 dB, the number of elements for convergence is higher and increases from 8 to 15. This is visible from Table 9.

A similar case is seen for variable parameters κ*_y_* and µ, and number of branches *L*, presented in Table 10. The number of additions increases from 5 to 16 for all values of used parameters.

### 3.2. Average Fade Duration

The AFD shows the average time that the signal envelope spends below the specified threshold level. This LCR measurement is used to design a diversity scheme for cellular systems. The AFD is expressed in units of seconds.

The AFD can be evaluated as the ratio of the Pout and the LCR ([35], Equation (2.106)):

(41) $$AFD=\frac{Pout}{N_{z}\left(z\right)}$$

When putting (15) and (40) into (41), the final expression for the AFD for the considered case is:

(42) $$AFD=\frac{\sum_{j_{1}=0}^{+\infty }\overset{+\infty }{\underset{j_{2}=0}{\sum }}\frac{{\left(\kappa_{x}m_{i}\right)}^{j_{1}}{\left(\kappa_{y}\mu_{i}\right)}^{j_{2}}}{j_{1}!j_{2}!}\frac{\Gamma \left(j_{1}+j_{2}+\mu_{i}+m_{i}\right)}{\Gamma \left(j_{1}+m_{i}\right)\Gamma \left(j_{2}+\mu_{i}\right)}B_{\frac{m_{i}s_{i}{z_{i}}^{2}}{\mu_{i}\Omega_{i}\left(1+\kappa_{y}\right)+m_{i}s_{i}{z_{i}}^{2}}}\left(j_{1}+m_{i},j_{2}+\mu_{i}\right)}{L\sqrt{2\pi }f_{m}\sum_{j_{3}=0}^{+\infty }\overset{+\infty }{\underset{j_{4}=0}{\sum }}\frac{{\kappa_{x}}^{j_{3}}\kappa_{y}^{j_{4}}{m_{i}}^{2j_{3}+m_{i}-\frac{1}{2}}{\mu_{i}}^{2j_{4}+\mu_{i}-\frac{1}{2}}{\left(\Omega_{i}\left(1+\kappa_{y}\right)\right)}^{j_{4}+\mu_{i}-\frac{1}{2}}{s_{i}}^{j_{3}+m_{i}-\frac{1}{2}}{z_{i}}^{2j_{3}+2m_{i}-1}\Gamma \left(j_{3}+j_{4}+m_{i}+\mu_{i}-1/2\right)}{j_{3}!j_{4}!\Gamma \left(j_{3}+m_{i}\right)\Gamma \left(j_{4}+\mu_{i}\right){\left(\mu_{i}\Omega_{i}\left(1+\kappa_{y}\right)+m_{i}s_{i}z_{i}^{2}\right)}^{j_{3}+j_{4}+m_{i}+\mu_{i}-1}}}$$

**Figure 14:**
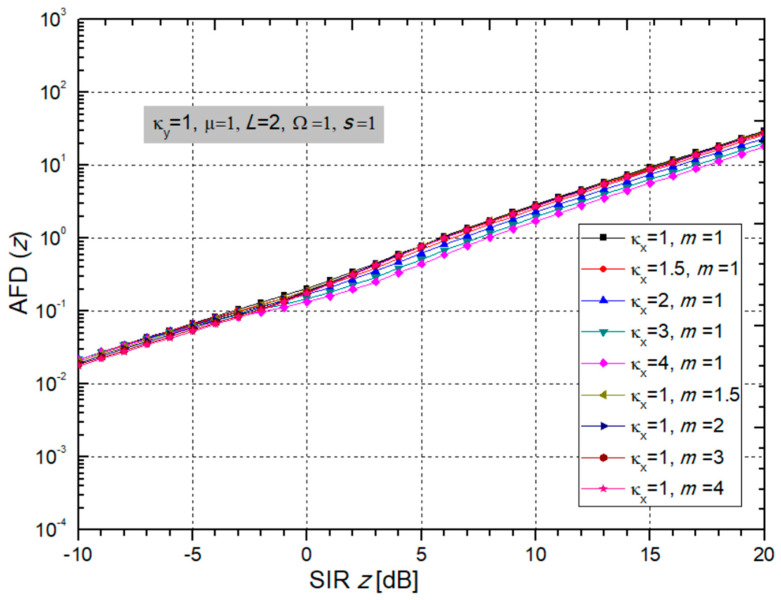
The AFD depending on output SIR for different values of BX fading parameters κ_x_ and m; while CCI parameters are: κ_y_ = 1 and µ = 1, number of branches L = 2 and powers: Ω = 1, s = 1.

**Figure 15:**
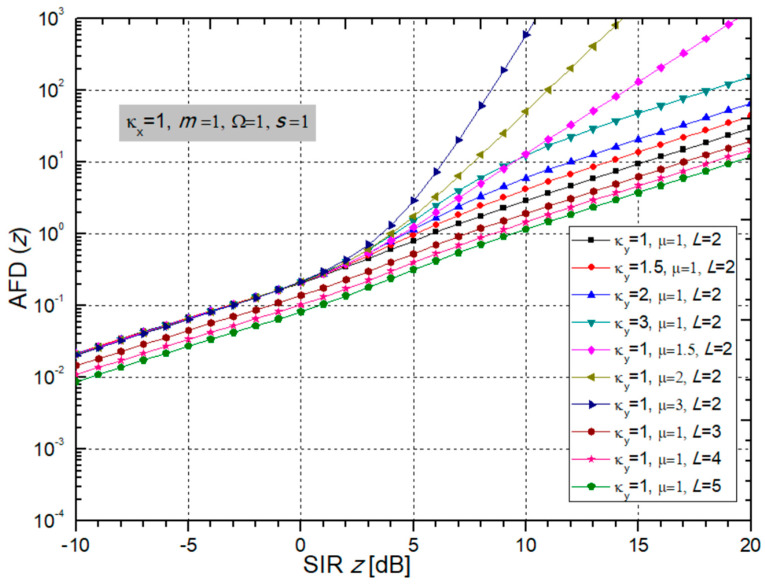
The AFD versus SIR considering different values of CCI parameters κ_y_ and µ and number of branches L, while BX fading parameters are: κ_x_ = 1 and m = 1, and powers Ω = 1 and s = 1.

The graphs for AFD are presented in the two Figure 14 and Figure 15. From Figure 14, it is obvious that BX fading parameters κ*_x_* and *m* do not affect the AFD much. When parameter *m* increases, so does the AFD, which is bad for system performance. When parameter κ_x_ increases, then the AFD decreases, and the system performance is better, resulting in a lower AFD.

On the other hand, the normalized curves for AFD presented in Figure 15 show that the AFD is less for a larger number of receiver input branches *L*, which facts improve the system performance. These are less difficult environments, as can be seen in Figure 15.

When the crossing threshold *z* is below the average signal level, the AFD is low, and this is generally the regime in which the system normally operates. When the Rician κ_y_ factor increases, there is interference, the power of the dominant LOS component increases or the power of the scattering components decreases, thus making the fading less pronounced. The system then has lower performance, and the AFD increases, but the AFD is not affected so much by the CCI parameter κ_y_. Also, an increase in parameter µ spoils the performance because it increases the AFD.

Below are tables with the number of additions in the sum in expression (42) needed to achieve the required precision on the fifth significant digit.

From Table 11, it can be seen that when parameter κ*_x_* is increasing, convergence and required accuracy are achieved when a maximum of 9 additions are added for *z* = −10 dB and *z* = 0 dB, while for *z* = 10 dB, the number of required elements increases, and the series in expression (42) converges more slowly.

When parameter *m* increases, the number of elements that need to be added to obtain the accuracy of expression (42) to the 5th decimal tends to be constant for *z* = −10 dB (increases from 6 to 8), while for *z* = 0 dB and *z* = 10 dB, the number of elements increases, and the series converges more slowly.

From Table 12, one can see that the number of additions in sums in (42) becomes bigger when κ*_y_* and µ increase, while this number of additions is quite small when *L* increases.

## 4. LLM- and MDE-Enabled Network Planning Workflow

The emerging Large Language Model (LLM)-based ChatGPT (https://chat.openai.com/, accessed on 8 April 2024) human-like conversational service has drawn significant attention in both industry and academia, which resulted in many novel adoptions and use cases in various areas, ranging from creative content writing to programming [35]. Taking into account the experiments carried out by curious researchers and enthusiasts around the world, it can be summarized that LLMs are able to cover various relevant aspects within the generation of computer applications and the software development process itself, as well. Among these adoptions, some of them, besides LLMs, rely on their synergy with model-driven engineering (MDE) [36], making many innovative usage scenarios possible [36,37,38]: (1) domain conceptualization—metamodel construction based on free-form textual information; (2) instance creation—metamodel and natural language text are used as inputs, while the target output is instance of a model with respect to that metamodel; (3) modeling constraint extraction—identification of rules that must hold within model instances, where inputs are these constraints in textual form, along with the given metamodel, while the outputs are formal logic rules, such as Object Constraints Language (OCL); (4) generation of code—code templates together with model instances are taken as inputs and used for the purpose of generating executable program code, targeting some specific platform or programming language.

Considering the previously mentioned LLM and MDE synergy use cases, in this paper, we adopt these techniques with the goal of reducing the overall cognitive load and effort needed for wireless network planning and experimentation. Due to the increasing complexity of infrastructure, besides the growing number of the involved devices and their heterogeneous nature, the process of next-generation network-related prototyping and experimentation is highly challenging task [36,38]. For that reason, in this paper, we used an approach leveraging MDE tools (Eclipse Ecore (https://eclipse.dev/modeling/emf/, accessed on 8 April 2024) and Object Constraints Language—OCL (https://www.omg.org/spec/OCL/2.4/About-OCL, accessed on 8 April 2024)) for representation of domain concepts and their relationships, together with constraints and, on the other hand, trending ChatGPT as LLM representative in order to enable automated creation of model instances based on input text, extract domain constraints from text and, finally, generate the experiment code based on model instances.

**Figure 16:**
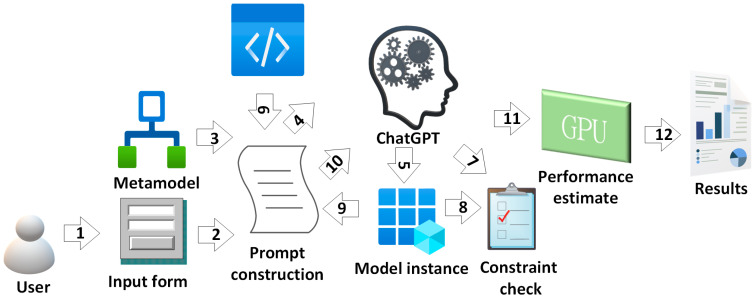
MDE and LLM synergy-based workflow for next-generation network experimentation and planning: 1—Natural language text experiment description and constraints; 2—Taking user-defined input to Prompt construction script; 3—Eclipse Ecore-based metamodel representation; 4—Prompt1 and Prompt 2 executions; 5—Model instance; 6—Experiment template; 7—Model instance as input for code generation; 8—OCL rules for verification of model instance; 9—Verified model instance; 10—Prompt3 execution; 11—Parametrized experiment; 12—Performance estimations, such as Pout, CC, ABEP, LCR, AFD.

In Figure 16, we depict the proposed workflow.

First, the user provides text describing the desired configuration of network planning experiment using natural language text, covering also constraints, such as design limitations and aspects related to performance. Apart from user input, a metamodel is also leveraged in order to construct two prompts targeting ChatGPT:

**Prompt** **1:** *According to {Experiment configuration description text} generate Ecore model instance based on given metamodel {Ecore metamodel}*

**Prompt** **2:** *According to {Experiment limitations text} generate OCL constraints based on given metamodel {Ecore metamodel}*

Furthermore, the script responsible for LLM prompt construction was implemented using Python programming language and OpenAI API (https://platform.openai.com/docs/api-reference, accessed on 8 April 2024) for ChatGPT. The result of the first prompt is model instance, which represents experiment configuration in a form compliant with the given metamodel. However, the outcome of the second prompt is a set of OCL rules which are checked if they hold against the model instance.

Once both the rules and model instance are extracted using LLM, a design-time consistency check of the model instance is performed in order to determine if all the given constraints hold. After that, taking into account the specified fading environment configuration, such as outage probability and channel capacity, performance-related elements are assigned to the instance model as well.

Additionally, in order to speed up such calculations, a GPU-enabled approach is adopted introducing loop-based computation parallelization, building upon our past works presented in [36,38]. With the aim to achieve this, the following prompt for parametrizing experiment script leveraging model instance is executed:

**Prompt** **3:** *Parametrize experiment based on template {experiment template} using model instance {model instance}*

**Figure 17:**
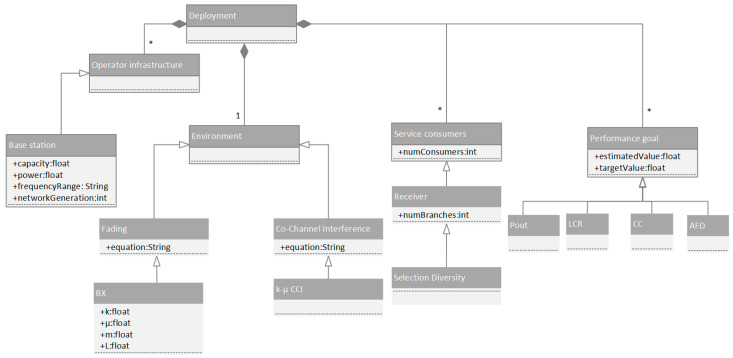
Network experimentation metamodel.

The elements of the underlying metamodel for experiment representation are depicted in Figure 17. Here, the cardinalities are denoted as: 1-single instance participates in relationship; *-multiple instances of same type within the relationship allowed. The top-level concept within the metamodel is deployment. Deployment consists of elements representing service provider infrastructure like base stations, and, on the other side, considers the end users of these services, while these users can rely on different types of receivers.

Regarding the elements of the underlying telco infrastructure, the properties such as power consumption, frequency range, and capacity expressed as number of active users and target network generation (2G–5G) are considered. Moreover, aspects expressing the environmental configuration are also considered as fading and co-channel interference types. In this context, we also consider distinct parameters, specific for the particular type of fading and co-channel interference model.

In the end, the proposed metamodel also covers performance-related goals, such as boundary values for channel capacity and outage probability. The estimated performance value is compared to these goals expressed in the form of OCL rules, so the user will be notified whether the experimental deployment is compliant with these requirements.

In what follows, Table 13 shows example OCL rules for the described scenario.

In Table 14, an overview of the results achieved for different experiment configurations is given, considering the execution time spent for relevant steps. Compared to our previous works proposing the workflow where knowledge of domain modeling tools was necessary, in this paper, the refined workflow requires significantly less time. Manually, around 12 min for a single experiment were needed, while the proposed workflow reduces to the order of magnitude of 10 s, so the experimentation workflow speed-up is significant.

The approach relying on an LLM-aided approach significantly reduces the time required for creation of a single experiment and overall cognitive overload, as only free-form text has to be provided by the end user of the planning tool.

## 5. Conclusions

The main contribution of our paper is modelling of a wireless channel with a multi-branch SC diversity receiver in the presence of Beaulieu-Xie fading and CCI with κ-µ distribution. There is no defined fading distribution in the literature that can adequately model the multiple specular signal components transmitting between the transmitter and the receiver together with the diffused components, which is a typical environment in the case of femtocells and high-speed trains. For the aforementioned reasons, we presented here derivations for different first- and second-order performances for such a model. Another great advantage of introducing the BX fading distribution is that the existing fading distributions (κ-µ, Nakagami-m, Rayleigh, Rician, etc.) can be obtained as its special cases by adjusting a combination of parameters. Since κ-µ is also general fading distribution, it is possible to apply the obtained results to a large number of system configurations in the presence of fading and CCI with different listed distributions.

Additionally, the presented approach making use of LLMs and MDE significantly reduces both the effort and time required for wireless network experimentation as it takes natural language text descriptions as input from the end user. This way, the required cognitive load is reduced as mastering additional tools for conceptual modeling directly is not required.

In our future work, we will analyze the performance of wireless systems in the presence of fading and CCI with these and other general distributions, although with EGC and MRC diversity receivers. Using EGC and MRC diversity receivers achieves better performance than using SC receivers, which is popular for its simplicity, affordability, and favorable price, but with some loss in performance quality.

## Figures and Tables

**Table 1:** Number of terms to be added in expression (15) for Pout to achieve accuracy at the 5th significant digit when changing parameters κ_x_ and *m*, and κ*_y_* = 1, µ = 1, Ω = 1, s = 1, *L* = 2.

Pout	*z* = −10 dB	*z* = 0 dB	*z* = 10 dB
κ_x_ = 1, *m* = 1	5	8	9
κ_x_ = 1.5, *m* = 1	5	7	10
κ_x_ = 2, *m* = 1	5	7	12
κ_x_ = 2.5, *m* = 1	5	7	12
κ_x_ = 3, *m* = 1	5	7	14
κ_x_ = 4, *m* = 1	5	8	16
κ_x_ = 1, *m* = 1.5	5	8	10
κ_x_ = 1, *m* = 2	5	8	11
κ_x_ = 1, *m* = 2.5	5	9	13
κ_x_ = 1, *m* = 3	5	10	14
κ_x_ = 1, *m* = 4	5	12	16

**Table 2:** Number of terms to be added in the expression for Pout (15) to achieve precision to the 5th significant digit for variables κ_y_, µ, and *L*. Other parameters are: κ*_x_* = 1, *m* = 1, Ω = 1, *s* = 1.

Pout	*z* = −10 dB	*z* = 0 dB	*z* = 10 dB
κ_y_ = 1, µ = 1, *L* = 2	5	8	9
κ_y_ = 2, µ = 1, *L* = 2	5	10	12
κ_y_ = 3, µ = 1, *L* = 2	5	13	13
κ_y_ = 4, µ = 1, *L* = 2	5	15	16
κ_y_ = 1, µ = 2, *L* = 2	5	11	12
κ_y_ = 1, µ = 3, *L* = 2	5	13	13
κ_y_ = 1, µ = 4, *L* = 2	5	15	16
κ_y_ = 1, µ = 1, *L* = 3	5	7	9
κ_y_ = 1, µ = 1, *L* = 4	5	7	9
κ_y_ = 1, µ = 1, *L* = 5	5	6	9
κ_y_ = 1, µ = 1, *L* = 2	5	8	9

**Table 3:** The number of terms in the sum in the expression for CC/B (20) in order to achieve precision on the 5th significant digit when changing the fading parameters κ_x_ and *m*. Other parameters are: κ*_y_* = 1, µ = 1, Ω = 1, s = 1, *L* = 2.

CC/B	*w_i_* = −10 dB	*w_i_* = 0 dB	*w_i_* = 10 dB
κ_x_ = 1, *m* = 1	8	9	10
κ_x_ = 1.5, *m* = 1	10	10	11
κ_x_ = 2, *m* = 1	11	12	12
κ_x_ = 2.5, *m* = 1	12	13	14
κ_x_ = 3, *m* = 1	14	15	14
κ_x_ = 4, *m* = 1	16	16	17
κ_x_ = 1, *m* = 1.5	9	10	10
κ_x_ = 1, *m* = 2	11	11	12
κ_x_ = 1, *m* = 2.5	12	13	13
κ_x_ = 1, *m* = 3	14	14	14
κ_x_ = 1, *m* = 4	16	16	17

**Table 4:** Number of additions in the sum in expression (20) for CC/B to reach accuracy at the 5th significant decimal for variable CCI parameters κ_y_ and µ, and number of branches *L.* The fading parameters and powers are: κ*_x_* = 1, *m* = 1, Ω = 1, s = 1.

CC/B	*w_i_* = −10 dB	*w_i_* = 0 dB	*w_i_* = 10 dB
κ_y_ = 1, µ = 1, *L* = 2	8	9	10
κ_y_ = 1.5, µ = 1, *L* = 2	10	10	10
κ_y_ = 2, µ = 1, *L* = 2	11	12	11
κ_y_ = 3, µ = 1, *L* = 2	13	14	14
κ_y_ = 1, µ = 1.5, *L* = 2	9	10	10
κ_y_ = 1, µ = 2, *L* = 2	10	11	11
κ_y_ = 1, µ = 3, *L* = 2	13	14	15
κ_y_ = 1, µ = 1, *L* = 3	8	9	9
κ_y_ = 1, µ = 1, *L* = 4	9	10	10
κ_y_ = 1, µ = 1, *L* = 5	9	9	9

**Table 5:** The number of additions in sum in expression (25) to achieve precision to the 5th significant digit for MGF-based ABEP for BFSK, when the fading parameters κ_x_ and *m* change, and others are: κ_y_ = 1 µ = 1, Ω = 1, s = 1, *L* = 2.

ABEP-BFSK	*w_i_* = −10 dB	*w_i_* = 0 dB	*w_i_* = 10 dB
κ_x_ = 1, *m* = 1	8	8	7
κ_x_ = 1.5, *m* = 1	9	8	7
κ_x_ = 2, *m* = 1	10	9	8
κ_x_ = 2.5, *m* = 1	11	11	8
κ_x_ = 3, *m* = 1	12	12	10
κ_x_ = 4, *m* = 1	15	14	11
κ_x_ = 1, *m* = 1.5	9	8	7
κ_x_ = 1, *m* = 2	11	10	8
κ_x_ = 1, *m* = 2.5	12	11	10
κ_x_ = 1, *m* = 3	12	12	10
κ_x_ = 1, *m* = 4	15	15	13

**Table 6:** The number of additions to be summed in expression (25) to achieve accuracy at the 5th significant digit for MGF-based ABEP for BDPSK; the CCI parameters κ_y_ and µ are variable, as well as number of branches *L*, and constant are: κ_x_ = 1, *m* = 1, Ω = 1, s = 1.

ABEP-BFSK	*w_i_* = −10 dB	*w_i_* = 0 dB	*w_i_* = 10 dB
κ_y_ = 1, µ = 1, *L* = 2	8	8	7
κ_y_ = 1.5, µ = 1, *L* = 2	10	9	8
κ_y_ = 2, µ = 1, *L* = 2	10	10	10
κ_y_ = 3, µ = 1, *L* = 2	14	13	12
κ_y_ = 1, µ = 1.5, *L* = 2	9	9	8
κ_y_ = 1, µ = 2, *L* = 2	11	10	9
κ_y_ = 1, µ = 3, *L* = 2	13	12	11
κ_y_ = 1, µ = 1, *L* = 3	8	8	7
κ_y_ = 1, µ = 1, *L* = 4	8	8	7
κ_y_ = 1, µ = 1, *L* = 5	8	8	7

**Table 7:** The number of additions in expression (26) to achieve accuracy at the 5th significant decimal for MGF-based ABEP for BDPSK. The fading parameters κ_x_ and *m* are variable, the CCI parameters are: κ*_y_* = 1, µ = 1, powers Ω =1, s = 1, and number of branches *L* = 2.

ABEP-BDPSK	*w_i_* = −10 dB	*w_i_* = 0 dB	*w_i_* = 10 dB
κ_x_ = 1, *m* = 1	8	8	6
κ_x_ = 1.5, *m* = 1	9	8	7
κ_x_ = 2, *m* = 1	10	8	6
κ_x_ = 2.5, *m* = 1	11	9	7
κ_x_ = 3, *m* = 1	12	10	8
κ_x_ = 4, *m* = 1	14	12	8
κ_x_ = 1, *m* = 1.5	9	8	6
κ_x_ = 1, *m* = 2	10	9	7
κ_x_ = 1, *m* = 2.5	11	10	8
κ_x_ = 1, *m* = 3	13	11	9
κ_x_ = 1, *m* = 4	14	14	10

**Table 8:** The number of additions have to be summed in (26) to achieve precision at the 5th significant digit for MGF-based ABEP for BDPSK; the CCI parameters κ_y_ and µ, and number of branches *L*, are variable; constants are fading parameters κ*_x_* = 1 and *m* = 1, and powers: Ω = 1 and s = 1.

ABEP-BDPSK	*w_i_* = −10 dB	*w_i_* = 0 dB	*w_i_* = 10 dB
κ_y_ = 1, µ = 1, *L* = 2	8	8	6
κ_y_ = 1.5, µ = 1, *L* = 2	9	9	7
κ_y_ = 2, µ = 1, *L* = 2	11	10	9
κ_y_ = 3, µ = 1, *L* = 2	13	13	11
κ_y_ = 1, µ = 1.5, *L* = 2	10	9	8
κ_y_ = 1, µ = 2, *L* = 2	10	10	9
κ_y_ = 1, µ = 3, *L* = 2	13	12	11
κ_y_ = 1, µ = 1, *L* = 3	8	7	6
κ_y_ = 1, µ = 1, *L* = 4	8	7	6
κ_y_ = 1, µ = 1, *L* = 5	8	7	5

**Table 9:** The number of required additions for summing in expression (40) to achieve precision at the 5th significant decimal for LCR; the fading parameters κ*_x_* and *m* change, the CCI parameters κ*_y_* and µ are constant: κ_y_ = 1, µ = 1 and number of branches *L* = 2, powers are: Ω = 1 and s = 1.

LCR	*z* = −10 dB	*z* = 0 dB	*z* = 10 dB
κ_x_ = 1, *m* = 1	5	9	8
κ_x_ = 1.5, *m* = 1	5	8	9
κ_x_ = 2, *m* = 1	5	8	11
κ_x_ = 2.5, *m* = 1	5	8	12
κ_x_ = 3, *m* = 1	5	9	13
κ_x_ = 4, *m* = 1	5	9	16
κ_x_ = 1, *m* = 1.5	5	8	9
κ_x_ = 1, *m* = 2	5	9	11
κ_x_ = 1, *m* = 2.5	5	9	12
κ_x_ = 1, *m* = 3	5	11	14
κ_x_ = 1, *m* = 4	5	13	15

**Table 10:** The number of additions have to be added in the sum in (40) to reach accuracy at the 5th significant digit for LCR; the CCI parameters κ_y_ and µ, and number of branches *L*, are variable; constant are fading parameters κ_x_ = 1 and *m* = 1, and powers: Ω = 1 and s = 1.

LCR	*z* = −10 dB	*z* = 0 dB	*z* = 10 dB
κ_y_ = 1, µ = 1, *L* = 2	5	9	8
κ_y_ = 2, µ = 1, *L* = 2	7	12	10
κ_y_ = 3, µ = 1, *L* = 2	9	14	12
κ_y_ = 4, µ = 1, *L* = 2	11	16	13
κ_y_ = 1, µ = 2, *L* = 2	7	11	8
κ_y_ = 1, µ = 3, *L* = 2	9	13	9
κ_y_ = 1, µ = 4, *L* = 2	11	16	10
κ_y_ = 1, µ = 1, *L* = 3	5	7	8
κ_y_ = 1, µ = 1, *L* = 4	5	7	8
κ_y_ = 1, µ = 1, *L* = 5	5	7	9

**Table 11:** Required number of terms need to be summed in expression (42) to achieve precision at the 5th significant digit for the AFD with variable fading parameters κ*_x_* and *m*, while the CCI parameters are unchanging: κ*_y_* = 1, µ = 1, number of branches *L* = 2, powers are: Ω = 1 and s = 1.

AFD	*z* = −10 dB	*z* = 0 dB	*z* = 10 dB
κ_x_ = 1, *m* = 1	7	8	7
κ_x_ = 1.5, *m* = 1	6	7	10
κ_x_ = 2, *m* = 1	7	7	11
κ_x_ = 2.5, *m* = 1	6	6	12
κ_x_ = 3, *m* = 1	7	8	13
κ_x_ = 4, *m* = 1	6	9	16
κ_x_ = 1, *m* = 1.5	7	7	10
κ_x_ = 1, *m* = 2	6	5	11
κ_x_ = 1, *m* = 2.5	7	9	13
κ_x_ = 1, *m* = 3	6	10	13
κ_x_ = 1, *m* = 4	8	12	15

**Table 12:** The number of additions in sums in (42) for reaching precision at the 5th significant digit for the AFD; the CCI parameters κ_y_ and µ, and number of branches *L*, are variable; permanent fading parameters are: κ*_x_* = 1 and *m* = 1, and powers: Ω = 1 and s = 1.

AFD	*z* = −10 dB	*z* = 0 dB	*z* = 10 dB
κ_y_ = 1, µ = 1, *L* = 2	7	8	7
κ_y_ = 2, µ = 1, *L* = 2	9	10	11
κ_y_ = 3, µ = 1, *L* = 2	10	13	14
κ_y_ = 4, µ = 1, *L* = 2	12	15	17
κ_y_ = 1, µ = 2, *L* = 2	9	11	12
κ_y_ = 1, µ = 3, *L* = 2	10	13	14
κ_y_ = 1, µ = 4, *L* = 2	12	14	16
κ_y_ = 1, µ = 1, *L* = 3	6	8	7
κ_y_ = 1, µ = 1, *L* = 4	6	8	7
κ_y_ = 1, µ = 1, *L* = 5	5	7	7

**Table 13:** Examples for generated OCL rules.

Text	OCL Rule
Deployment should have at least two base stations	context Deployment inv deploymentHasAtLeastTwoBaseStations: self.baseStations->size() >= 2
Outage probability of deployment should be less than 0.05	context Deployment inv outageProbabilityBelowThreshold: self.outageProbability < 0.05
Minimal number of service consumers supported should be 150	context Deployment inv MinimumServiceConsumers: self.serviceConsumers.numConsumers >= 150

**Table 14:** LLM-enabled workflow evaluation approach.

Aspect	Manual Efforts	Execution Time [s] 1 Receiver 2 Receivers	Experiment Description
Text to model instance	50 s—sentence typing	8.4 13.2	Beaulieu-Xie fading κ-µ CCI, diversity combining outage probability 1 receiver/2 receivers
Model instance to experiment	Automatic	4.3 9.6	
Performance estimation	Automatic	1.8 2.9	
Constraint definition	30 s—sentence typing	7.9 12.6	

## Data Availability

Data are contained within the article. Auxiliary processing scripts and metamodel are publicly available on GitHub: https://github.com/penenadpi/chatgpt_ecore_ocl.
